# Selected Histone Deacetylase Inhibitors Reverse the Frataxin Transcriptional Defect in a Novel Friedreich’s Ataxia Induced Pluripotent Stem Cell-Derived Neuronal Reporter System

**DOI:** 10.3389/fnins.2022.836476

**Published:** 2022-02-23

**Authors:** Anna M. Schreiber, Yanjie Li, Yi-Hsien Chen, Jill S. Napierala, Marek Napierala

**Affiliations:** ^1^Department of Biochemistry and Molecular Genetics, Stem Cell Institute, University of Alabama at Birmingham, Birmingham, AL, United States; ^2^Genome Engineering and iPSC Center, Washington University, St. Louis, MO, United States

**Keywords:** Friedreich’s ataxia (FRDA), reporter cell line, induced pluripotent stem cells, neural progenitor cells (NPCs), Nanoluciferase, screening

## Abstract

Friedreich’s ataxia (FRDA) is a neurodegenerative disorder caused by the expansion of guanine–adenine–adenine repeats within the first intron of the frataxin (*FXN*) gene. The location and nature of the expansion have been proven to contribute to transcriptional repression of *FXN* by decreasing the rate of polymerase II (RNA polymerase II) progression and increasing the presence of histone modifications associated with a heterochromatin-like state. Targeting impaired *FXN* transcription appears as a feasible option for therapeutic intervention, while no cure currently exists. We created a novel reporter cell line containing an *FXN*-Nanoluciferase (*FXN-NLuc*) fusion in induced pluripotent stem cells (iPSCs) reprogrammed from the fibroblasts of patients with FRDA, thus allowing quantification of endogenous *FXN* expression. The use of iPSCs provides the opportunity to differentiate these cells into disease-relevant neural progenitor cells (NPCs). NPCs derived from the FXN-NLuc line responded to treatments with a known *FXN* inducer, RG109. Results were validated by quantitative PCR and Western blot in multiple FRDA NPC lines. We then screened a commercially available library of compounds consisting of molecules targeting various enzymes and pathways critical for silencing or activation of gene expression. Only selected histone deacetylase inhibitors were capable of partial reactivation of *FXN* expression. This endogenous, FRDA iPSC-derived reporter can be utilized for high-throughput campaigns performed in cells most relevant to disease pathology in search of *FXN* transcription activators.

## Introduction

Friedreich’s ataxia (FRDA; OMIM, #229300) is an autosomal recessive neurodegenerative disorder, affecting approximately 1:20,000–1:50,000 people of the Caucasian population (Bürk, 2017; Indelicato et al., 2020). It is the most common inherited ataxia (Marmolino, 2011; Cook and Giunti, 2017) with a typical onset at 10–15 years of age (Parkinson et al., 2013). FRDA is described as a multisystemic disease, manifesting in peripheral and central nervous systems, with a significant group of patients affected by cardiomyopathy, diabetes, and hearing and vision impairment (Koeppen, 2011; Delatycki and Corben, 2012). The molecular pathology of FRDA is driven by the large trinucleotide guanine–adenine–adenine (GAA) expansion in the first intron of the frataxin (*FXN*) gene, located on chromosome 9q13-q21.1 (Campuzano et al., 1996). For the majority of patients, an expansion is present on both *FXN* alleles (Dürr et al., 1996), with a smaller population harboring a single expanded GAA tract accompanied by a point mutation (Cossée et al., 1999; Clark et al., 2017). Healthy individuals carry up to 38 GAA repeats, whereas hyperexpansion in patients with FRDA can exceed 1,000 repeats (Pandolfo, 2009). FXN is a mitochondrial protein involved in iron–sulfur cluster (Fe–S) synthesis and overall iron metabolism (Martelli et al., 2007). Deficiency of FXN in FRDA leads to a broad spectrum of molecular aberrations including but not limited to mitochondrial iron accumulation, increased oxidative stress, and reactive oxygen species (ROS) formation (Clark et al., 2018).

The consequence of GAA repeat expansion is transcriptional silencing of the *FXN* gene, resulting in low levels of FXN (Bidichandani et al., 1998; Ohshima et al., 1998; Dion and Wilson, 2009). Two major, not mutually exclusive, mechanisms leading to *FXN* transcription inhibition have been proposed: formation of non-B DNA and/or DNA–RNA structures (Bidichandani et al., 1998) and localized chromatin changes (Saveliev et al., 2003). The formation of non-canonical DNA structures such as intramolecular triplexes or R-loops (Bergquist et al., 2016) leads to defects in transcription initiation (De Biase et al., 2009) and decreased rate of RNA polymerase II (RNAPII) elongation (Li et al., 2015a). At the same time, differences in histone modifications at the *FXN* locus, typically immediately upstream and downstream of the expanded repeats, between FRDA and unaffected controls have been recurrently observed (Herman et al., 2006). These include decreased acetylation of histones H3 and H4 (H3K9ac and H4K5ac) and increased histone H3 trimethylation at lysines 9 and 27 (H3K9me3 and H3K27me3) as well as elevated DNA cytosine methylation, all demonstrated in cellular and mouse FRDA models (Herman et al., 2006; Greene et al., 2007; Al-Mahdawi et al., 2008; Punga and Bühler, 2010; Yandim et al., 2013). Combined, data from numerous studies point toward DNA/DNA–RNA conformation(s)-induced chromatin changes as a trigger for *FXN* downregulation.

Targeting impaired *FXN* transcription in FRDA represents one of the main therapeutic strategies for this devastating disease (Gottesfeld, 2019; Zhang et al., 2019). Numerous small molecules aimed to reverse the repressive chromatin environment at the *FXN* locus have been tested (Sarsero et al., 2003; Herman et al., 2006; Sandi et al., 2014; Georges et al., 2019). *FXN* expression is upregulated by treatment with histone deacetylase inhibitors (HDACi) (Bürk, 2017), especially RG109 (also known as RG2833) (Soragni et al., 2014; Chutake et al., 2016; Codazzi et al., 2016), nicotinamide, and resveratrol (Chan et al., 2013; Li et al., 2013; Georges et al., 2019). In addition, treating FRDA cells with histone methyltransferase (HMT) inhibitors that reduce H3K9me2/3, H3K27me3 (Sherzai et al., 2020), and H4K20me2/3 (Vilema-Enríquez et al., 2020) demonstrated modest increases in *FXN* expression, indicating a role for histone methylation also in repressing *FXN* transcription.

*FXN* reactivation was also observed after targeting GAA tracts (or structures formed by these repeats) using chemically modified single- and double-stranded antisense oligonucleotides (ASOs) (Li L. et al., 2016; Li et al., 2018; Shen et al., 2018) or pyrrole-imidazole polyamides (PAs) (Burnett et al., 2006; Gottesfeld, 2007). Combining GAA-specific PAs with a small-molecule ligand engaging transcription elongation machinery resulted in a novel type of hybrid compound termed Synthetic Transcription Elongation Factor 1 (SynTef1), also capable of stimulating *FXN* transcription (Erwin et al., 2017). Lastly, several other compounds, including interferon gamma, antioxidants, and nuclear factor erythroid 2–related factor 2 (Nrf2) activators, were shown to increase FXN levels although their mechanisms of action have not been clearly defined (Gottesfeld, 2019).

Some of the described transcription activators have reached early phases of clinical trials (Gottesfeld, 2019); however, identification of novel lead compounds capable of stimulating *FXN* expression in FRDA cells is laborious and time consuming. To aid this process, several reporter cell lines designed to mimic the *FXN* transcription defect while allowing for robust evaluation of numerous compounds have been utilized (Grant et al., 2006; Soragni et al., 2008; Li et al., 2013; Lufino et al., 2013). Initial FRDA reporter cell lines were designed on the basis of engineered HeLa (Grant et al., 2006) or HEK293 FlpIn (Soragni et al., 2008) cells harboring a green fluorescent protein (GFP) gene with expanded GAA tracts. Neither of these reporter systems directly reported endogenous FXN levels. Next-generation reporters addressed this issue by using bacterial artificial chromosome-based constructs carrying the entire human *FXN* gene modified by an in-frame insertion of a reporter gene (enhanced GFP or luciferase) (Li et al., 2013; Lufino et al., 2013). These constructs were integrated in the genomes of HEK293 (Lufino et al., 2013) or HeLa (Li et al., 2013) cells (not at the *FXN* locus) and harbor short or moderately expanded GAA repeat tracts.

Recent studies indicate that both long-range interactions and locations of repeat-containing genes in the genome may be important for their functions (Sun et al., 2018; Ruiz Buendía et al., 2020). Thus, an FRDA reporter cell line, to better reflect physiological regulatory mechanisms, should be engineered at the endogenous *FXN* locus in its natural chromosomal/genomic context. It should also harbor expanded GAA repeats of lengths representative of the patient population, and it would be of benefit to create an FRDA reporter system using cells that represent tissues/organs primarily affected by the disease.

To fulfill all these criteria and to efficiently screen for compounds capable of reversing GAA-induced transcriptional silencing of *FXN*, we generated a novel reporter line using cells derived from a patient with FRDA, thus enabling the investigation of endogenous FXN levels impacted by long GAA repeats. We performed a knock-in of a Nanoluciferase (NLuc) reporter gene into the *FXN* locus in induced pluripotent stem cells (iPSCs) reprogrammed from patient fibroblasts. These iPSCs were further differentiated into neural progenitor cells (NPCs) and neurons. As a proof of concept, we screened a library containing 281 compounds targeting different pathways involved in chromatin maintenance and transcription regulation. Our luciferase-based screen validated the reporter as a reliable tool in search of novel inducers of *FXN* expression. Results of our analyses revealed HDACi as the only class of compounds represented in the library capable of reproducible reactivation of *FXN* expression.

## Materials and Methods

### Reprogramming Friedreich’s Ataxia Fibroblasts to Induced Pluripotent Stem Cells

FRDA fibroblasts containing expanded GAA repeats and control fibroblast cell lines were obtained from the Friedreich’s Ataxia Cell Line Repository (FACLR) established at the Napierala laboratory at the University of Alabama at Birmingham (UAB) in collaboration with Dr. David Lynch from Children’s Hospital of Philadelphia (CHOP) with appropriate approvals of the Institutional Review Boards (IRBs; CHOP IRB no. 10-007864; UAB IRB no. N131204003) (Li Y. et al., 2016). Skin biopsies were donated by patients with FRDA and unaffected individuals for derivation of primary fibroblast lines. All biopsy samples (and resulting cell lines) are de-identified. Fibroblasts were transduced with Sendai virus vectors, encoding the four reprogramming factors, namely, octamer-binding transcription factor 3/4 (Oct3/4), cellular myelocytomatosis oncogene (c-Myc), kruppel like factor 4 (Klf4), and sex determining region Y-Box 2 (Sox2), using the CytoTune iPS 2.0 Sendai Reprogramming Kit (Life Technologies) to generate iPSCs. Cells were cultured on Matrigel^®^-coated plates (Corning) with mTeSR medium (STEMCELL Technologies). Medium changes were performed daily with passages every 5–7 days with Dispase (STEMCELL Technologies) and 10 μM ROCK inhibitor (Stemgent) as described by Polak et al. (2016). Analyses of pluripotency, differentiation potential, and karyotype were performed as described by Li et al. (2015b). A description of iPSC lines used in this study can be found in Supplementary Table 1.

### Knock-in of Nanoluciferase Using CRISPR/Cas9

**FIGURE 1:**
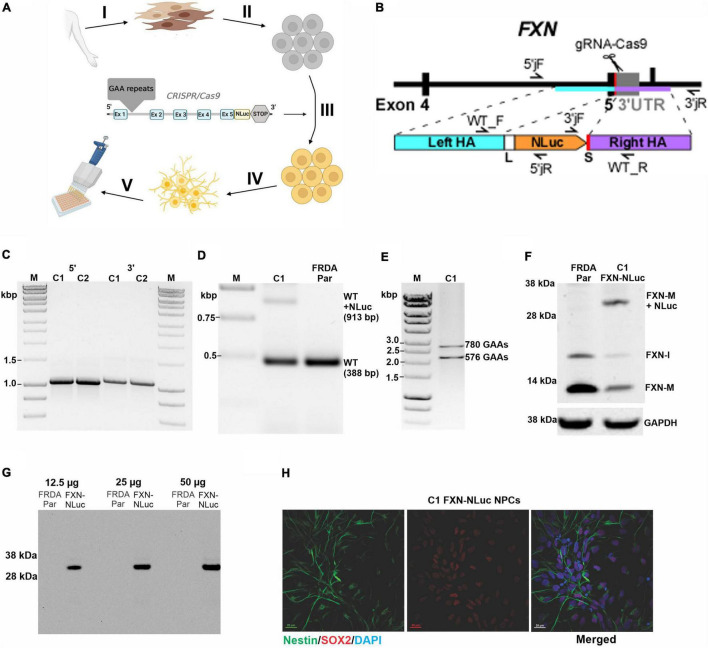
Generation and characterization of the FXN-NLuc reporter. **(A)** Schematic overview of FXN-NLuc reporter cell line generation. Primary fibroblasts of patients with FRDA were derived from a forearm skin biopsy (I) and reprogrammed to iPSCs (II). An in-frame fusion between the *FXN* gene and *NLuc* gene was generated *via* CRISPR/Cas9 genome editing (III). FXN-NLuc iPSCs were differentiated into NPCs (IV), generating a ready-to-use reporter cell line for compound screening (V). Image was created with BioRender.com. **(B)** Schematic presentation of the CRISPR/Cas9-mediated editing strategy used to generate the *FXN-NLuc* fusion. Approximate locations of the gRNA as well as primers used for confirmation of the editing are presented. **(C)** Validation of 5′ and 3′ junctions by PCR in two knock-in iPSC clones (C1 and C2). The 5′ junction was amplified using 5′jF and 5′jR (expected product size 1,068 bp); the 3′junction was amplified using 3′jF and 3′jR (expected product of 1,100 bp). M, size marker. **(D)** Confirmation of heterozygous knock-in of the *NLuc* gene in FRDA iPSCs. PCR was performed using primers WT_F and WT_R. C1 represents an edited clone [both unedited (WT) allele of 388 bp and edited WT + NLuc allele of 913 bp are amplified]; FRDA Par represents the unedited FRDA parental line. M, size marker. **(E)** Agarose gel electrophoresis of PCR products confirming the presence of expanded GAA repeats in edited FRDA iPSCs (clone C1 is shown). Primers amplify the GAA repeat tract and additional flanking sequences of 498 bp; M, size marker. **(F)** Western blot analysis of FXN and FXN-NLuc fusion expression in parental, unedited FRDA iPSCs (FRDA Par) and edited cells (C1, FXN-NLuc), FXN-M + NLuc (∼32 kDa)–fusion protein. GAPDH serves as a loading control. **(G)** In-gel detection of the luminescent FXN-NLuc fusion protein. Increasing amounts of cell lysates prepared from parental (FRDA Par) and edited (FXN-NLuc) iPSCs were separated by SDS-PAGE and the luminescent signal was detected using the Nano-Glo^®^ In-Gel Detection System. **(H)** Differentiation of FXN-NLuc iPSCs into Nestin and Sox2 positive NPCs. Scale bar, 20 mm.

One million parental FRDA iPSCs were transfected with 2 μg of pX330-U6-Chimeric_BB-CBhhSpCas9 (Addgene, #42230) encoding SpCas9 and expressing a gRNA targeting *FXN* downstream of the stop codon using a Human Stem Cell Nucleofector Kit (Lonza). Three days after nucleofection, single cells were seeded at clonal density in mTeSR medium supplemented with CloneR (STEMCELL Technologies) and cultured for 4 days. Colonies were picked manually after 10–14 days. After expansion, genomic DNA was isolated from individual clones and screened for the presence of the correct 5′ and 3′ junctions using primers listed below. In addition, the locations of primers, gRNA binding sequence, and the exact amino acid sequence of the C-terminus of FXN fused with NLuc are shown in Figure 1 and Supplementary Figure 1. The polymerase chain reactions (PCRs) for 5′ and 3′ junctions and to detect the presence of the wild-type (WT), unedited allele were performed with 100 ng of DNA with the REDTaq^®^ ReadyMix™ PCR Reaction Mix (Sigma-Aldrich) using the following cycling conditions: initial denaturation step at 94°C for 2 min, followed by 30 cycles of 30 s at 94°C, 30 s at 60°C, and 2 min at 72°C, and a final extension step of 5 min at 72°C. Expected sizes of the amplicons are described in the legend of Figure 1. Long GAA repeats were amplified using 100 ng of genomic DNA with the FailSafe™ PCR 2X PreMix D and FailSafe™ PCR Enzyme Mix (Epicenter) with primers spanning 498 base pairs (bp) of flanking GAA sequences (Li et al., 2015b) using the following cycling conditions: initial denaturation step for 3 min at 94°C, followed by 20 cycles of 20 s at 94°C, 30 s at 64°C, and 5 min at 68°C, followed by additional eight cycles of 20 s at 94°C and 5 min at 68°C, with 15-s increase of every elongation step and a final extension step of 7 min at 68°C. Sequences of primers (5′–3′) used for *FXN-NLuc* reporter DNA sequence validation: 5′ junction: 5′j forward: GTTTAGGTGATTGCTGGGGTGC3′; 5′j reverse: CCAACGAAATCTTCGAG TGTGA; 3′ junction: 3′j forward: GTTCCGAGTAACCATCAACGGAG; 3′j reverse: CCCTCATCTACCCAAT AGGGAAT; detection of unedited allele: WT_forward: TGGTTCATCTGAAGGGCTGTGCTGT GG; WT_reverse: TGGGGGCAAGGTAGGAGGCAACACA; GAA repeats: GAA_forward: GGCTTGAACTTCCCACACG TGTT; GAA_reverse: AGGACCATCATGGCCACACTT.

### Differentiation of Neural Progenitor Cells

NPCs were differentiated using the STEMdiff™ SMADi Neural Induction Kit (STEMCELL Technologies) as monolayer cultures, according to the manufacturer’s recommendation. Two million iPSCs per well were plated on Matrigel^®^-coated six-well plates (Corning) in a single-cell suspension using STEMdiff™ Neural Induction Medium + SMADi (STEMCELL Technologies), supplemented with 10 μM ROCK inhibitor (Stemgent). Daily medium changes were performed with STEMdiff™ Neural Induction Medium + SMADi (STEMCELL Technologies). Cells were passaged after 7 days using Accutase (STEMCELL Technologies) and plated at a density of 2 × 10^6^ cells per well. After 7 days of growth, cells were passaged for a total of two passages. Mature NPCs were then plated at a density of 1.2 × 10^6^ cells per well on Matrigel^®^-coated six-well plates (Corning) and cultured in STEMdiff™ Neural Progenitor Medium (STEMCELL Technologies) with daily medium changes.

### Generation of Terminally Differentiated Neurons

Neuronal differentiation was performed as described by Zhang et al. (2013), with modifications, using lentiviral vectors for Ngn2 (ys13-002 plasmid) and rtTA (ys13-003 plasmid) overexpression. On the initial day, 1 × 10^6^ iPSC cells per well were plated on Matrigel^®^-coated six-well plates (Corning) in a single-cell suspension using mTeSR medium (STEMCELLTechnologies) with thiazovivin (BioVision). After 24 h, cells were infected with rtTA-Ub and Ngn2 viruses, in dulbecco’s modified eagle medium: nutrient mixture F-12 (DMEM/F12) medium (Gibco) supplemented with Polybrene (MilliporeSigma). Twenty-four hours after transfection, the culture medium was replaced with DMEM/F12 (Gibco), supplemented with N-2 (Life Technologies), non-essential amino acids (NEAA) (Invitrogen), human brain derived neurotrophin factor (BDNF) (PeproTech), human neurotrophin 3 (NT-3) (PeproTech), and mouse laminin (Invitrogen). To induce TetO expression, doxycycline (Clontech) was added to the medium. One day after induction of TetO expression, the medium was changed to Neurobasal (Gibco) supplemented with B-27 (Thermo Fisher Scientific), GlutaMAX (Invitrogen), human BDNF (PeproTech), human NT-3 (PeproTech), and doxycycline (Clontech). Puromycin (EMD Millipore) was added to the supplemented medium to start cell selection. Cells were cultured in Neurobasal supplemented medium for 12 days, with daily medium changes. After 2 weeks of differentiation, cells were treated with Accutase (STEMCELL Technologies) and plated onto poly-D-lysine (Sigma)/laminin (Invitrogen) coated plates, at a density of 2 × 10^6^ cells per well. After re-plating, cortical neurons were cultured for 7 days in supplemented Neurobasal medium, with half of the medium changed every 2 days.

### Luciferase Assays

Luciferase assays were performed using Nano-Glo^®^ Luciferase Assay System (Promega) according to the manufacturer’s recommendation. The FXN-NLuc NPCs were plated on 96-well Matrigel-coated (Corning) white plates in triplicate, at a density of 2.5 × 10^4^ cells per well. Cells were treated with compounds in different concentrations (as indicated in the “Results” section) and cultured in STEMdiff™ Neural Progenitor Medium (STEMCELL Technologies) for 24, 48, or 72 h depending on experimental design. Nano-Glo^®^ Luciferase Assay Substrate was mixed with Nano-Glo Luciferase Assay Buffer in a proportion of 1:50. Subsequently, Nano-Glo^®^ Luciferase Assay Reagent was added to FXN-NLuc NPCs at a volume equal to the culture media and mixed. Luminescence was measured using FLUOstar Omega plate reader (BMG Labtech) with blank media and Nano-Glo^®^ Luciferase Assay Reagent as a background control.

### In-Gel Luciferase Assays

To verify the size of the FXN-NLuc fusion protein and identify any potential non-specific NLuc expression, in-gel luciferase assays were performed. Cell lysates were prepared using Passive Lysis Buffer (Promega) supplemented with protease inhibitor cocktail (Sigma-Aldrich). Protein concentration was determined using a Bradford Protein Assay kit (Bio-Rad). Whole-cell extracts (12.5, 25, and 50 μg) along with the SeeBlue Plus2 Pre-stained Protein Standard (Fisher Scientific) were electrophoresed on NuPAGE 4–12% Bis-Tris gels (Life Technologies). Gels were washed with 25% isopropanol, rinsed with double-distilled water, then covered in Nano-Glo^®^ In-Gel Detection Reagent (Promega), and incubated for 10 min. The luminescent signal was detected with Nano-Glo^®^ In-Gel Detection System (Promega) and visualized using a ChemiDoc MP Imaging System (Bio-Rad).

### Epigenetics Compound Library Description and Screening

The luciferase-based screening was performed using DiscoveryProbe™ Epigenetics Compound Library (APExBIO). The compounds were diluted to 10 μM concentration in dimethyl sulfoxide (DMSO) (Sigma-Aldrich). FXN-NLuc NPCs were plated on 96-well white plates at a density of 2.5 × 10^4^ cells per well. Treatments were performed for 24 h. Screening was performed in duplicate using two independently cultured batches of FXN-NLuc NPCs with DMSO as a vehicle control. Luminescence detection was conducted as described above.

Half maximal effective concentration values for selected compounds were calculated using GraphPad Prism 9 software.

### RNA Isolation and Quantitative Real-Time PCR

All RNA samples were isolated using the RNeasy Plus Kit (QIAGEN), followed by DNase treatment with Turbo DNase (Thermo Fisher Scientific). Quantitative real-time PCR (qRT-PCR) reactions were performed using 50 ng of RNA with the iTaq™ Universal SYBR^®^ Green One-Step Kit (Bio-Rad) on a CFX Opus 96 Real-Time PCR Instrument (Bio-Rad). *FXN* transcript levels were normalized to glyceraldehyde 3-phosphate dehydrogenase (*GAPDH*) or hypoxanthine phosphoribosyltransferase 1 (*HPRT1*) and quantified relative to the respective control sample using the ΔΔCt method. The following primers (shown as 5′–3′) were used in qRT-PCR analyses: *FXN* forward: CAGAGGAAACGCTGGACTCT; *FXN* reverse: AGCCAGATTTGCTTGTTTGG; *GAPDH* forward: GAAGGTGAAGGTCGGAGTC; *GAPDH* reverse: GAAGATGG TGATGGGATTTC; *HPRT1* forward: TGACACTGGCAAAAC AATGCA, *HPRT1* reverse: GGTCCTTTTCACCAGCAAGCT.

### Protein Isolation and Western Blot

Protein isolation and Western blot were performed as described by Li et al. (2019). Briefly, cell lysates were prepared in whole-cell extraction buffer containing 0.1% IGEPAL CA-630 (IGEPAL) CA-630 (NP-40), 0.25 M sodium chloride (NaCl), 5 mM edetic acid (EDTA), and 50 mM 4-(2-hydroxyethyl)piperazine-1-ethanesulfonic acid (pH 7.5) supplemented with protease inhibitor cocktail (Sigma-Aldrich). Protein concentration was determined using a Bradford Protein Assay kit (Bio-Rad). Fifty micrograms of protein extracts were electrophoresed on NuPAGE 4–12% Bis-Tris gel (Life Technologies) followed by protein transfer onto 0.2 mM nitrocellulose membrane (Bio-Rad). Human FXN protein was detected using anti-FXN antibody diluted to 1:1,000 (ProteinTech Group) incubated overnight at 4°C. Membranes were then incubated with anti-rabbit Immunoglobulin G (IgG) conjugated to horseradish peroxidase (HRP) (GE Healthcare) diluted to 1:7,500 for 1 h at room temperature. Human GAPDH protein was detected using anti-GAPDH antibody (Sigma-Aldrich) diluted to 1:10,000 and incubated for 1 h at room temperature. Human HPRT1 protein was detected using anti-HPRT1 antibody (Cell Signaling Technology) diluted to 1:1,000 and incubated for 1 h at room temperature. Membranes were then incubated with anti-mouse IgG conjugated to HRP (GE Healthcare) diluted to 1:7,500 for 1 h at room temperature. Signals were visualized using Thermo Scientific™ SuperSignal™ West Femto Maximum Sensitivity Substrate (Life Technologies) using a ChemiDoc MP Imaging System (Bio-Rad) and quantified using ImageLab software (Bio-Rad).

### Immunocytochemistry

NPCs markers SOX2 and Nestin were detected by imaged-based immunocytochemistry analyses. Cells were plated onto Matrigel-coated (Corning) 12-well plates on cover slips at a density of 0.4 × 10^6^ cells per well. After 2 days, cells were fixed using 4% paraformaldehyde, permeabilized with 0.1% Triton-X in phosphate-buffered saline (PBS), and blocked with 2.5% normal donkey serum (Abcam) in PBS/Tween 20. SOX2 protein was detected using anti-SOX2 antibody (ProteinTech Group) diluted to 1:500, and Nestin protein was detected using anti-Nestin antibody (Sigma-Aldrich) diluted to 1:200, following 1-h incubation at room temperature. Cells were incubated with Alexa Fluor 488 antibody (Thermo Fisher Scientific) diluted to 1:2,000 for Nestin detection and with Alexa Fluor 568 antibody (Thermo Fisher Scientific) diluted to 1:1,000 for SOX2 detection at room temperature for 45 min. Prepared coverslips were mounted onto slides using ProLong™ Gold Antifade Mountant with 4′,6-diamidino-2-phenylindole (DAPI) (Invitrogen) and imaged using a Nikon A1R confocal microscope (UAB High Resolution Imaging Facility).

### Lactate Dehydrogenase Cytotoxicity Assay

Cytotoxicity assays were performed using the CyQUANT™ Lactate Dehydrogenase (LDH) Cytotoxicity Assay (Invitrogen) according to the manufacturer’s recommendation. Cells were seeded in 96-well tissue culture plates in triplicate in STEMdiffperformed using the Neural Progenitor Medium (STEMCELL Technologies) at a density of 2.5 × 10^4^ cells per well. Besides compound-treated samples, two assay controls were added: untreated cells (spontaneous LDH activity) and lysed cells (maximum LDH activity). Fifty microliters of medium from each sample was transferred to a flat bottom 96-well plate and supplemented with 50 μl of LDH Cytotoxicity assay reaction mixture (containing assay substrate in assay buffer prepared according to the manufacturer’s recommendations), followed by 30 min incubation in the dark. Subsequently, 50 μl of Stop Solution was added before absorbance measurements at 490 and 680 nm were taken using a FLUOstar Omega plate reader (BMG Labtech). The A680 nm was subtracted from A490 nm as background before the calculations. Relative cytotoxicity was expressed as follows:


$$\%\mathrm{of}\mathrm{cytotoxicity}=\frac{\begin{array}{c} \left(\mathrm{compound}-\mathrm{treated}\mathrm{LDH}\mathrm{activity}\mathrm{sample}\right)-\left(\mathrm{spontaneous}\mathrm{LDH}\mathrm{activity}\right)\; \end{array}}{\left(\mathrm{maximum}\mathrm{LDH}\mathrm{activity}\right)-\left(\mathrm{spontaneous}\mathrm{LDH}\mathrm{activity}\right)}*100$$

### Statistical Analyses

Statistical analyses and graphs were performed using unpaired two-tailed Student’s *t*-tests and one-way ANOVA in GraphPad Prism 9 software. The significance of the differences between groups of datasets was set to the following *p*-values: *p < 0.05, ^**^p < 0.01, and ^***^p < 0.001.

## Results

### Generation of Endogenous Frataxin-Nanoluciferase Fusion as a Novel Friedreich’s Ataxia Reporter Cell Line

A schematic overview of generation of the novel *FXN-NLuc* reporter cell line is shown in Figure 1A. FRDA iPSCs reprogrammed from patient fibroblasts were edited using CRISPR/Cas9 to site-specifically knock-in the NLuc gene at the 3′ end of exon 5 of endogenous *FXN* (Figure 1B and Supplementary Figure 1). A five-amino acid-long linker was inserted between the last FXN amino acid (Ala) and the first amino acid of NLuc (Val). Technical details are provided in section “Materials and Methods” and Supplementary Figure 1. Using three sets of primers, we confirmed both 5′ and 3′ junctions (Figure 1C and Supplementary Figure 1) as well as heterozygosity of the knock-in (Figure 1D). The sequence of the junction between *FXN* and *NLuc* was also confirmed by DNA sequencing (data not shown). We did not identify homozygous knock-in of *NLuc*, indicating that the FXN-NLuc fusion protein is likely not functional. Two independent clones (C1 and C2) were obtained, and all subsequent studies described herein were performed using clone C1. This line harbored two expanded GAA tracts of ∼780 and 580 repeats (Figure 1E) and Western blot analysis demonstrated expression of both unmodified FXN (∼13 kDa) and FXN-NLuc fusion (∼32 kDa) (Figure 1F). Although the possibility of random integration and expression of the promoterless NLuc DNA construct is low, we analyzed total protein extracts from the FXN-NLuc line for the presence of a non-specific NLuc signal using an in-gel luciferase assay. We detected only a single band at ∼32 kDa corresponding to the molecular weight of the FXN-NLuc fusion protein (Figure 1G). As iPSCs do not represent a disease-relevant cell type, we differentiated them to NPCs. These dividing cells demonstrate neuronal characteristics and can be easily passaged, cryopreserved, and expanded to large numbers necessary for HTS campaigns. Differentiated FXN-NLuc NPCs expressed *Nestin* and *Sox2* as determined by immunostaining (Figure 1H).

### Validation of the Frataxin-Nanoluciferase Neural Progenitor Cell Reporter Using a Known Frataxin Expression Inducer

**FIGURE 2:**
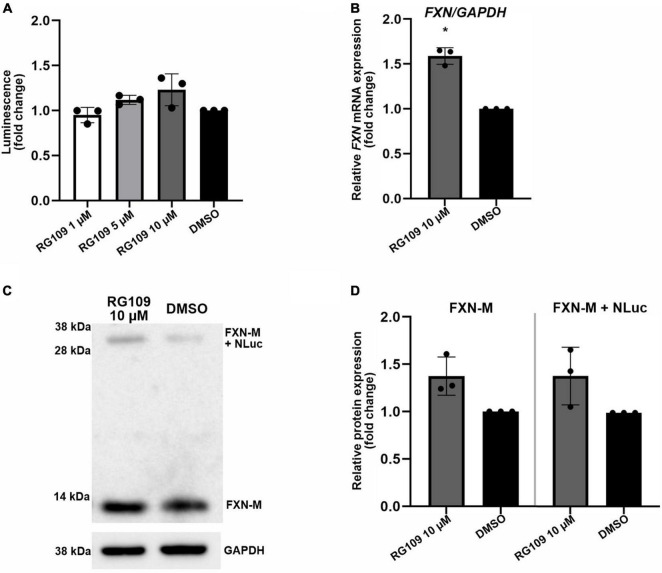
Validation of FXN-NLuc reporter NPCs with known *FXN* inducer RG109. **(A)** Increase of FXN-NLuc expression (luminescence) by HDACi RG109 treatment of NPCs for 24 h at 1, 5, and 10 μM. Results are shown relative to DMSO control. **(B)** qRT-PCR determination of *FXN* mRNA and *FXN-NLuc* mRNA expression upon 24-h treatment of NPCs with 10 μM HDACi RG109. Primers used for amplification are located in exons 3 and 4 of *FXN*. DMSO treatment served as a vehicle control. **(C)** Representative results of Western blot analyses of FXN-NLuc NPCs treated with 10 μM RG109 for 24 h. DMSO was used as a control. Endogenous mature FXN (FXN-M, ∼13 kDa) and FXN-NLuc fusion (FXN-M + NLuc, ∼32 kDa) are indicated. GAPDH served as a loading control. **(D)** Western blot quantitation. FXN-M and FXN-M + NLuc were quantified and plotted independently. Statistical calculations were performed using one-way ANOVA (non-parametric Kruskal–Wallis test) for data in **(A)** or Student’s *t*-test for data in **(B,D)**; *p < 0.05. Unless otherwise indicated, results represent mean ± *SD* from three independent experiments.

To test the reporter cell line utility, the FXN-NLuc cells were subjected to treatments with a known inducer of *FXN* expression, HDACi RG109. RG109 increased expression of *FXN-NLuc* in a concentration-dependent manner; however, the activation was modest and never reached statistical significance (Figure 2A). At the same time, 10 μM RG109 significantly increased *FXN* mRNA by ∼1.5-fold as determined by qRT-PCR (Figure 2B). Similar to luminescence, the assessment of the effect of RG109 treatment on FXN and FXN-NLuc protein expression using Western blot revealed slight but not significant increases (Figures 2C,D).

These results indicate that the FXN-NLuc reporter responds to a known *FXN* expression activator and can be used as a reliable model to identify new compounds capable of reactivating *FXN* in FRDA cells. Importantly, the response level of the reporter signal (luminescence) is comparable to the FXN and FXN-NLuc increase measured at the protein level using Western blot (Figure 2). Moreover, when measured separately, the response of the unmodified *FXN* allele is identical to the signal increase observed from the *FXN-NLuc* allele, indicating preservation of the *FXN* regulatory mechanisms at the stages of transcription, translation, and protein stability.

### Screening of Epigenetic Compound Library Identified Frataxin Expression Activator Candidates

**FIGURE 3:**
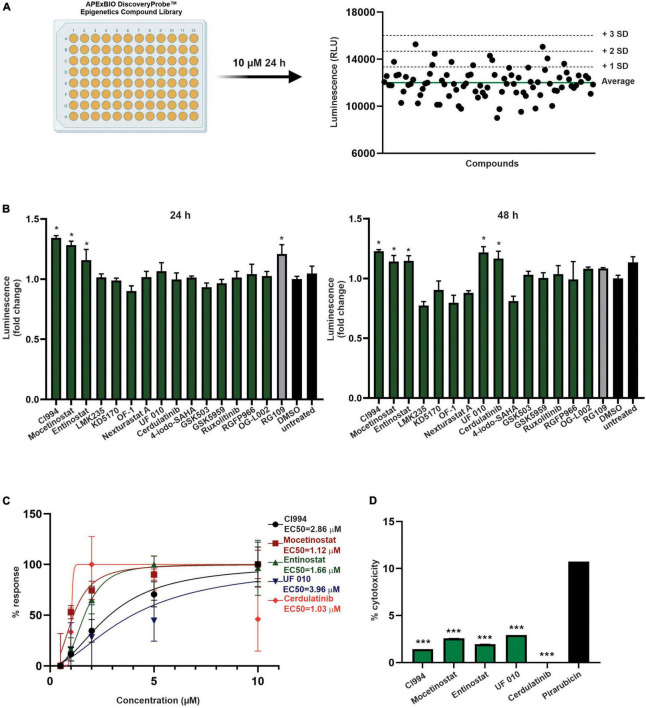
Screening of epigenetic compound library to identify novel inducers of *FXN* expression. **(A)** An overview of the screening process. All compounds of the APExBIO DiscoveryProbe™ Epigenetics Compound Library were tested at 10 μM concentration. Luminescence signal was plotted independently per each plate. The green line corresponds to plate average signal; dashed lines indicate average signal plus 1, 2, and 3 standard deviations of the mean. All data from two rounds of screening are included as Supplementary Figure 2. **(B)** Secondary validation of the efficacy of 16 compounds selected by initial screens. Luminescence detection was performed after 24 and 48 h of treatment. DMSO- and RG109-treated FXN-NLuc NPCs were used as controls. Five compounds were selected for further studies: CI994, Mocetinostat, Entinostat, UF 010, and Cerdulatinib. Results are mean ± SD from three technical replicates; * indicates *p* < 0.05 by one-way ANOVA. **(C)** Dose–response analyses for the selected compounds. Luminescence analyses were performed after 48 h of treatment with compounds at 0.5, 1, 2, 5, and 10 μM. EC50 is indicated for each compound. **(D)** Determination of cytotoxicity in FXN-NLuc NPCs using an LDH assay. Cytotoxicity is calculated relative to the spontaneous LDH release detected in DMSO-treated cells. Cells were treated with 10 μM of each compound for 48 h. Pirarubicin served as a positive control for cytotoxicity. Results are mean ± *SD* from three independent experiments; *** indicates *p* < 0.001 by one-way ANOVA.

Next, as a proof of concept, we performed a luciferase-based screening of the Discovery Probe Epigenetic Compound Library (APExBIO) that includes 281 compounds predominantly targeting various epigenetic and chromatin pathways (Supplementary Table 2). Treatment of FXN-NLuc NPCs was conducted at 10 μM concentration of the compounds for 24 h in a 96-well format (Figure 3A and Supplementary Figure 2), with DMSO-treated cells used as the vehicle control. Two independent screening rounds were performed. A total of 16 compounds were identified as candidates for further evaluation based on the following criteria: (i) luminescence signal greater than the average of all samples on a plate (library was screened on four separate plates) plus one standard deviation of the mean and (ii) consistent increase of the luminescence signal in two independent screening rounds (Table 1).

**TABLE 1:** Compounds identified in the screen of the APExBIO DiscoveryProbe™ Epigenetics Compound Library to increase expression of FXN-NLuc in FRDA NPCs.

Name	Pathway/Target
Cl994	HDAC inhibitor
Mocetinostat	HDAC inhibitor
Entinostat	HDAC inhibitor
RG109	HDAC inhibitor
LMK235	HDAC inhibitor
KD5170	HDAC inhibitor
Nexturstat	HDAC inhibitor
UF 010	HDAC inhibitor
4-iodo-SAHA	HDAC inhibitor
RGFP966	HDAC inhibitor
OF-1	Bromodomain inhibitor
GSK5959	Bromodomain inhibitor
Ruxolitinib	JAK inhibitor
Cerdulatinib	Syk/JAK inhibitor
OG-L002	LSD1 inhibitor
GSK503	EZH2 inibitor

One of the initial 16 upregulators of *FXN-NLuc* expression was RG109, confirming previously reported results and further validating the reporter line. The remaining hits represented HDACi (9), bromodomain inhibitors (2), janus kinase (JAK) and Syk/JAK inhibitors (2), and unique LSD1 as well as EZH2 inhibitors (Table 1). To confirm whether selected compounds indeed increase *FXN-NLuc* expression, additional assays were conducted with 24- and 48-h treatments (Figure 3B). Five compounds increased FXN-NLuc luminescence signal following either 24- or 48-h treatments: CI994, Entinostat, Mocetinostat, UF 010, and Cerdulatinib. Three of them (CI994, Entinostat, and Mocetinostat) stimulated reporter expression at both time points. We focused further studies on these five compounds.

### Efficacy and Toxicity of Candidate Compounds

To determine the most effective concentration of each compound, we performed dose–response analyses within the range of 0.5–10 μM. Cerdulatinib, Mocetinostat, and Entinostat demonstrated the lowest half maximal effective concentration (EC50) values (∼1.0, 1.1, and 1.7 μM, respectively), while CI994 and UF 010 required higher concentrations (∼2.9 and 4.0 μM, respectively) to increase *FXN-NLuc* expression (Figure 3C). Next, to determine whether the selected compounds exert cytotoxic effects, we treated FXN-NLuc NPCs for 48 h at 10 μM concentration and then performed cytotoxicity assays by measuring LDH release to the media. No overt toxicity of the compounds was detected relative to vehicle-treated control (Figure 3D).

### Histone Deacetylase Inhibitors Are Capable of Increasing Frataxin mRNA and Protein Expression in Friedreich’s Ataxia Neural Progenitor Cells

**FIGURE 4:**
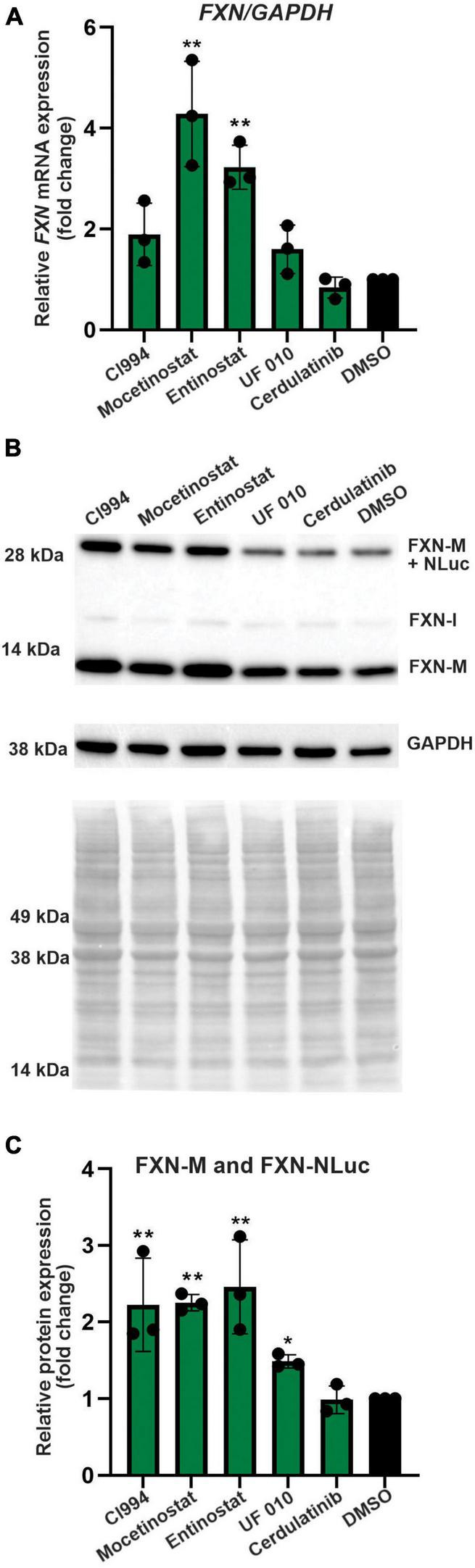
Selected HDACi increase FXN transcript and protein in FXN-NLuc NPCs. **(A)** Results of qRT-PCR analysis of *FXN* mRNA expression upon 48-h treatment with 10 μM CI994, Mocetinostat, Entinostat, UF 010, and Cerdulatinib. Results represent mean ± SD from three independent experiments; one-way ANOVA, **p < 0.01. DMSO treatment served as a control. **(B)** FXN and FXN-NLuc protein levels determined after treatment with indicated compounds for 48 h at 10 μM. Signals corresponding to mature FXN (FXN-M), intermediate FXN isoform (FXN-I), and FXN-NLuc fusion (FXN-M + NLuc) are indicated. GAPDH and Ponceau S staining served as loading controls. **(C)** Quantification of FXN and FXN-NLuc protein expression relative to Ponceau S staining. Cumulative signal from FXN and FXN-NLuc is shown. Results represent mean ± SD from three independent experiments; *p < 0.05 and **p < 0.01 determined by one-way ANOVA.

**FIGURE 5:**
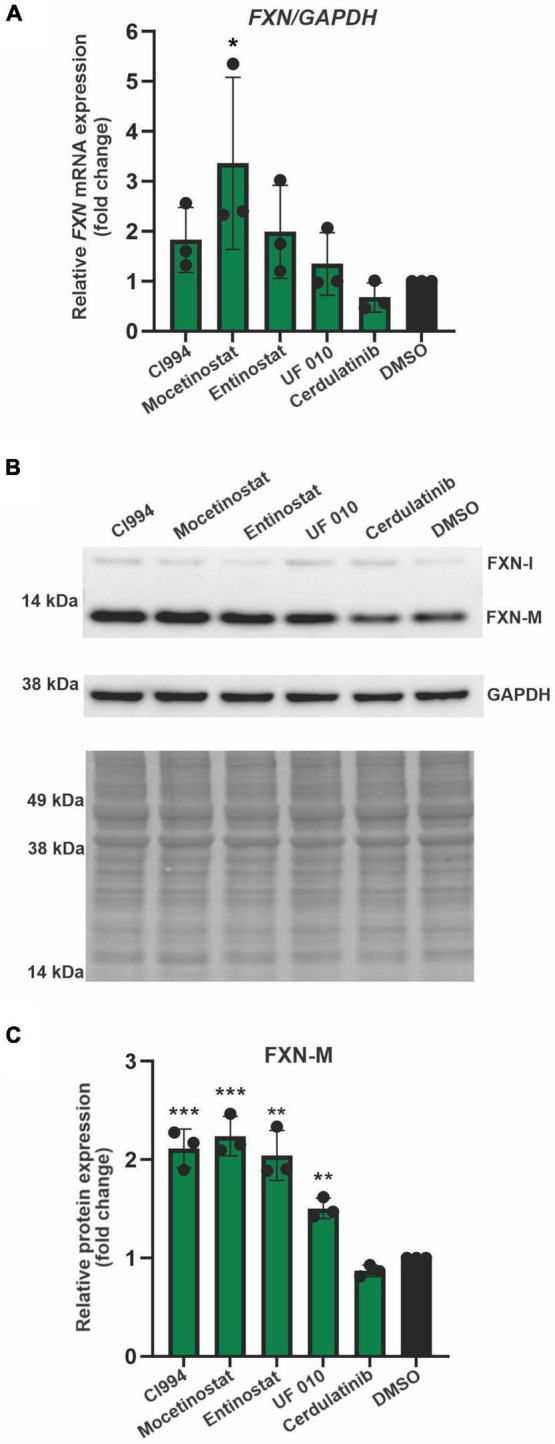
Validation of efficacy of identified HDACi in additional FRDA NPC lines. **(A)** Results of qRT-PCR analysis of *FXN* mRNA expression upon 48-h treatment with 10 μM CI994, Mocetinostat, Entinostat, UF 010, and Cerdulatinib. Results represent mean ± *SD* from three independent experiments conducted using three different NPC lines; *p < 0.05 determined by one-way ANOVA. DMSO treatment served as a control. **(B)** FXN protein expression determined after treatment with indicated compounds for 48 h at 10 μM. Signals corresponding to mature FXN (FXN-M) and intermediate FXN (FXN-I) are indicated. GAPDH and Ponceau S staining served as loading controls. A representative Western blot for the FRDA NPC lines tested is shown. **(C)** Quantification of FXN-M levels relative to Ponceau S staining. Results represent mean ± *SD* from three independent experiments obtained using three different FRDA NPC lines; **p < 0.01 and ***p < 0.001 determined by one-way ANOVA.

Next, we performed secondary validation of the five hits identified by the screen by qRT-PCR and Western blot using FXN-NLuc NPCs treated with 10 μM of the compounds. *FXN* transcript levels were increased following Mocetinostat and Entinostat treatment and, to a lesser extent, upon treatment with CI994 and UF 010 when compared to DMSO control (Figure 4A). Similarly, we detected ∼2–2.5-fold increase of the mature FXN protein and FXN-NLuc fusion (Figures 4B,C). Cerdulatinib failed to upregulate FXN expression at the transcript or protein levels. These results were confirmed in two additional FRDA NPC lines derived from different patient iPSCs (Figure 5). Although variable upregulation of *FXN* mRNA by CI994, Mocetinostat, and Entinostat treatments was determined, a consistent ∼2-fold increase in FXN protein was observed (Figures 5B,C). In addition, treatment with HDACi UF 010 resulted in ∼1.5-fold increase of FXN protein. In summary, testing in three independent FRDA NPC lines confirmed the efficacy of three HDACi identified by our reporter screen.

To determine whether the effect of the compounds is specific to FRDA cells, we also conducted analyses of *FXN* expression in control NPCs derived from an unaffected individual. CI994 and Mocetinostat treatment demonstrated ∼1.5-fold upregulation of *FXN* mRNA (Supplementary Figure 3A). A moderate (∼1.5-fold) upregulation of the FXN protein level was also detected upon treatment with CI994 and Entinostat when compared to DMSO control (Supplementary Figures 3B–D). Overall, tested compounds increased FXN levels in control cells to a lesser extent than in FRDA NPCs likely due to already higher basal *FXN* expression in the unaffected cells.

### Histone Deacetylase Inhibitors Reactivate Frataxin Expression in Terminally Differentiated Neurons

**FIGURE 6:**
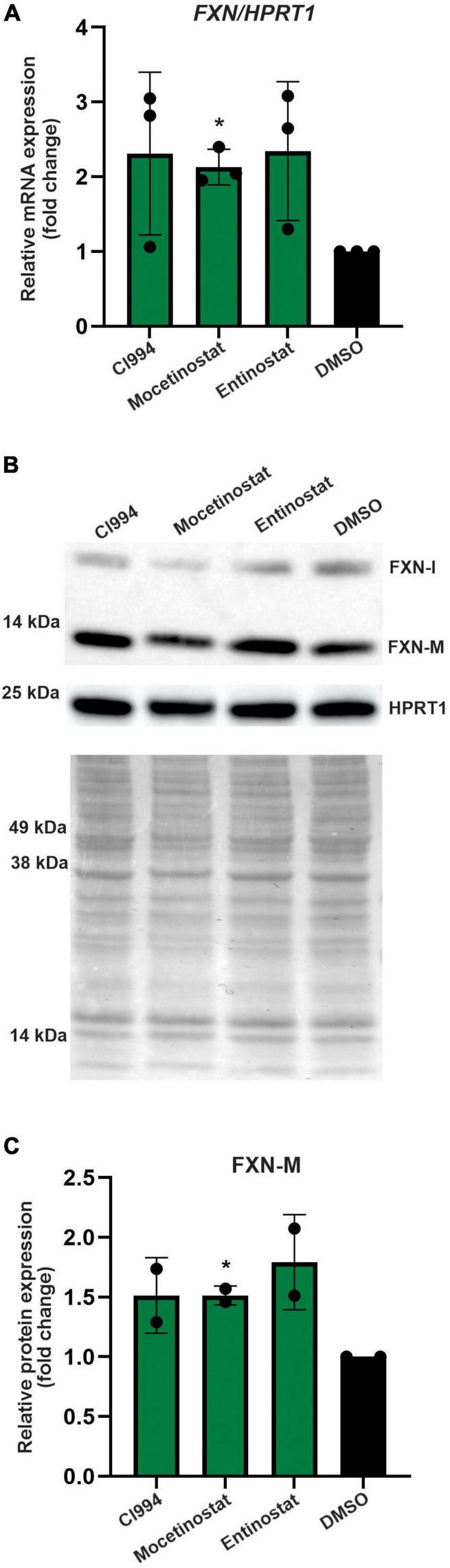
Validation of the most potent identified HDACi in terminally differentiated FRDA neurons. **(A)** Results of qRT-PCR analysis of *FXN* mRNA expression upon 48-h treatment with 10 μM CI994, Mocetinostat, and Entinostat. Results represent mean ± *SD* from three independent experiments conducted using three different FRDA neuronal lines; *p < 0.05 determined by independent Student’s *t*-test. DMSO treatment served as a control. **(B)** FXN protein expression determined after treatment with indicated compounds for 48 h at 10 μM concentration. The signals corresponding to mature FXN-M and FXN-I are indicated. HPRT1 and Ponceau S staining served as loading controls. A representative Western blot is shown. **(C)** Quantification of FXN-M levels relative to Ponceau S staining. Results represent mean ± *SD* from two independent experiments obtained using two different FRDA neuronal lines; *p < 0.01 determined by independent Student’s *t*-test.

Thus far, all results were obtained in proliferating NPCs. To determine whether the HDACi identified in our screen are capable of FXN upregulation in non-dividing cells, we tested the efficacy of CI994, Entinostat, and Mocetinostat in terminally differentiated neurons. The FRDA iPSC lines used for NPC differentiation were differentiated to cortical neurons (Zhang et al., 2013) and then treated with HDACi. The qRT-PCR results demonstrated, similarly to NPCs, that all three HDACi can upregulate *FXN* mRNA by ∼ 2–3-fold compared to the DMSO control (Figure 6A). We were also able to detect ∼1.5–1.75-fold increase of the mature FXN protein, confirming the efficacy of the three selected HDACi (Figures 6B,C). This concordance of results suggests that NPCs, which are significantly more amenable to large screening campaigns, represent a good surrogate for mature neuronal cells.

## Discussion

In this work, we designed, generated, and validated a novel FRDA reporter cell line derived from patient iPSCs that were modified by CRISPR/Cas9 to incorporate the *NLuc* gene fused to the terminal exon 5 of endogenous *FXN*. The reporter contains expanded GAA repeats within their natural genomic context of the *FXN* gene and enables luminescence-based detection of the FXN-NLuc fusion protein. FXN-NLuc reporter upregulation correlated well with both qRT-PCR and Western blot measurements of FXN mRNA and protein. The FXN-NLuc cell line is, to our knowledge, the first FRDA reporter based on patient-derived cells harboring long GAA repeats. Previously utilized reporter systems were predominantly derived from HeLa (Grant et al., 2006; Li et al., 2013) or HEK293 cells (Soragni et al., 2008; Lufino et al., 2013). Differentiation of iPSCs into NPCs and neurons (and potentially any other cell type of interest) presents an opportunity for testing the efficacy of *FXN* upregulation in disease-relevant cells or organoids. This flexibility represents an important advantage of iPSC-based reporters over systems created in fully committed cells from the perspective of variable efficacy of candidate compounds in different cell types. Recent studies on nicotinamide and resveratrol demonstrated their lack of efficacy toward *FXN* upregulation in terminally differentiated neurons (Georges et al., 2019) despite prior demonstration of *FXN* stimulation in fibroblasts and lymphocytes (Chan et al., 2013; Li et al., 2013), thus emphasizing the importance of conducting drug discovery research in disease-relevant cell types.

The molecular cause of FRDA has been known for 25 years and a role for chromatin involvement for almost 20 years. However, despite numerous efforts and screening campaigns, only a few small molecules capable of modest re-activation of *FXN* expression have been identified. A clear efficacy of specific HDACi (e.g., RG109) has been recurrently demonstrated in various model systems (Sandi et al., 2014; Soragni et al., 2014; Chutake et al., 2016; Codazzi et al., 2016). Targeting the HMT G9a affected histone methylation at *FXN* but did not increase its expression in FRDA lymphoblasts and fibroblasts (Punga and Bühler, 2010; Sherzai et al., 2020). On the other hand, a recent study targeting G9a and EZH2 HMTs reported, in addition to changes in H3K9me2/me3 and H3K27me3 levels, an increase of *FXN* mRNA expression in FRDA fibroblasts but without noticeable effects on FXN protein levels (Sherzai et al., 2020). This may be a result of an FRDA-specific patient cell response, as the length of the GAAs may influence the efficacy of some small molecules (Punga and Bühler, 2010). Inhibition of SUV420H1 (lysine methyltransferase 5B, KMT5B) by the small-molecule A-196 resulted in an FRDA-specific ∼1.5- to 2-fold upregulation of FXN levels *via* targeting H4K20me2/me3 (Vilema-Enríquez et al., 2020). Thus, despite numerous efforts, efficacious small-molecule-mediated reactivation of *FXN* expression in FRDA cells proves to be a challenging task.

The broad spectrum of chromatin and epigenetic processes targeted by the compounds utilized in this study as well as prior works by numerous laboratories raises a question regarding the role of chromatin changes in the molecular pathology FRDA. The small molecules tested in our study represent compounds targeting HDACs, HMTs, histone demethylases, bromodomains, and DNA methyltransferases, to name a few major classes (Supplementary Table 2). Out of this range of targets, predominantly, class 1, benzamide HDACi: CI994 (HDAC1 specific), Mocetinostat (pan HDACi), and Entinostat (class 1 HDACi) were capable of reproducible *FXN* upregulation in NPCs and terminally differentiated neurons. All three of them are potent HDAC inhibitors, developed and tested in clinical trials for various malignancies (Bondarev et al., 2021).

One possible explanation of relative inefficiency of chromatin manipulation in restoring *FXN* expression is that epigenome changes are secondary consequences of a physical barrier imposed by the expanded GAAs. Hence, without correcting the underlying issue at the DNA level, correcting the chromatin landscape is rather transient and inefficient. The relatively high efficacy of HDACi, demonstrated in this work and by numerous prior studies, may rely on the proximity of the GAA region to the promoter. Increased histone acetylation may facilitate the recruitment of RNAPII or promote an efficient transition from a paused state to productive elongation. In either of these scenarios, increased transcription of *FXN* would result from a higher rate of transcription through the gene rather than more efficient traversing of the expanded GAA repeats by the transcription machinery. That would also imply that a single compound targeting chromatin may not be completely efficacious in restoring *FXN* levels to asymptomatic carrier levels, especially in severe FXN deficiency cases caused by the longest GAA repeats. Although any upregulation of FXN levels may be of therapeutic significance (e.g., to delay disease progression), curative effects are likely to be achieved by ∼3- to 10-fold increase, depending on the length of the GAA tracts and correlated *FXN* repression observed in a given patient. Therefore, a combinational approach by targeting expanded GAA repeat tracts (e.g., with ASOs) and a small molecule targeting repressive chromatin may be required. In addition, hybrid molecules (e.g., SynTef1) combining GAA targeting with transcriptional modulation may represent an alternative as they could help reduce risks of non-specific effects of targeting enzymes involved in global control of gene expression.

## Conclusion

In conclusion, despite challenges associated with developing a small-molecule therapy to increase *FXN* expression, a patient cell-based reporter system utilizing endogenous *FXN* expression as a readout represents an excellent model to evaluate any pre-clinical approach aimed at increasing FXN mRNA or protein levels.

## Data Availability Statement

The original contributions presented in the study are included in the article/Supplementary Material, further inquiries can be directed to the corresponding author/s.

## Author Contributions

AS, YL, and Y-HC performed all experiments and analyzed the data. AS, YL, Y-HC, JN, and MN designed the experiments. AS, JN, and MN wrote the article. JN and MN acquired funding. All authors read the article, contributed comments and suggestions, and approved the final version of the article.

## Conflict of Interest

The authors declare that the research was conducted in the absence of any commercial or financial relationships that could be construed as a potential conflict of interest. The handling editor declared a past co-authorship with several of the authors AS, YL, JN, and MN.

## Publisher’s Note

All claims expressed in this article are solely those of the authors and do not necessarily represent those of their affiliated organizations, or those of the publisher, the editors and the reviewers. Any product that may be evaluated in this article, or claim that may be made by its manufacturer, is not guaranteed or endorsed by the publisher.
