# Supplementary material for: Selected Histone Deacetylase Inhibitors Reverse the Frataxin Transcriptional Defect in a Novel Friedreich’s Ataxia Induced Pluripotent Stem Cell-Derived Neuronal Reporter System
Source: Front Neurosci. 2022 Feb 23;16:836476. doi: 10.3389/fnins.2022.836476 (PMC8904878; doi:10.3389/fnins.2022.836476)
Supplement: Supplementary file 1 [file Data_Sheet_1.pdf]

Supplementary Material

Selected histone deacetylase inhibitors reverse the Frataxin transcriptional defect in a novel Friedreich's ataxia induced pluripotent stem cell-derived neuronal reporter system

Anna M. Schreiber[1], Yanjie Li[1], Yi-Hsien Chen[2], Jill S. Napierala[1], Marek Napierala*[1]

[1] Department of Biochemistry and Molecular Genetics, Stem Cell Institute, University of Alabama at Birmingham, Birmingham, AL, USA

[2]Genome Engineering and iPSC Center, Washington University, St Louis, MO, USA

Supplementary Figure 1 page 2

Supplementary Figure 2 page 6

Supplementary Figure 3 page 7

Supplementary Table 1 page 8

Supplementary Table 2 page 9

Supplementary Figure 1: DNA sequence of a fragment of the *FXN* gene. A fragment of *FXN* is shown along with the *NLuc* gene and the five amino acids linker between the coding sequences. Exact locations of the gRNA and all primers used in PCR amplifications to validate correct NLuc integration are shown. An overview of the strategy is presented in Figure 1.

```
5'    agacatggcagaagccagcctccgatctgtccacacgaata
...                                                                                      41,040
3'    tctgtaccgtcttcggtcggaggctagacaggtgtgcttat

cagcattcccaaagcaacgtgcatgtgccattattcactggatgagcttgaggtggatgaactagcccaccaggctctcaatgtcatgaa
                                                                                         41,130
gtcgtaagggtttcgttgcacgtacacggtaataagtgacctactcgaactccacctacttgatcgggtggtccgagagttacagtactt

                                                  5'jF
                                         gtttaggtgattgctggggtgc
tttaacactgaattaagaaaaatatgttttaaaaataatagtttaggtgattgctggggtgctaggagaggaaggaatggggaataactg
                                                                                         41,220
aaattgtgacttaattctttttatacaaaatttttattatcaaatccactaacgaccccacgatcctctccttccttaccccttattgac

tttaatgggtatagttggccttgtgtatctgtgggttccacatctgattcaaccaaccgtggatcaaaatatttgaaaataaaaaacaaa
                                                                                         41,310
aaattacccatatcaaccggaacacatagacacccaaggtgtagactaagttggttggcacctagttttataaactttatttttttgttt

acaaaaatgatacaaataaaaaccaatataacaactattaacagcatttacattgtactaggcattataagtaatctggagatgacttaa
                                                                                         41,400
tgtttttactatgtttatttttggttatattgttgataattgtcgtaaatgtaacatgatccgtaatattcattagacctctactgaatt

agcatacagaaggatgtgcctaggttagatgcatgtatcgtaccatttcatatcagggacttgagtacccacggattttggtatctgcag
                                                                                         41,490
tcgtatgtcttcctacacggatccaatctacgtacatagcatggtaaagtatagtccctgaactcatgggtgcctaaaaccatagacgtc

atcctggaaccccttccctatggataccaaggaacaacagcactgggtctccttttggggtgatgcagatgttttgaagctaggcagagg
                                                                                         41,580
taggaccttggggaagggatacctatggttccttgttgtcgtgacccagaggaaaaccccactacgtctacaaaacttcgatccgtctcc

tagtggttgcacaacattgtaaatgtactaaatgccaccaaattattcattttaaatggttaatgtgttatgtgaatttcaccttaaca
                                                                                         41,670
atcaccaacgtgttgtaacatttacatgatttacggtggtttaataagtaaaaatttaccaattacacaatacacttaaagtggaattgt
```

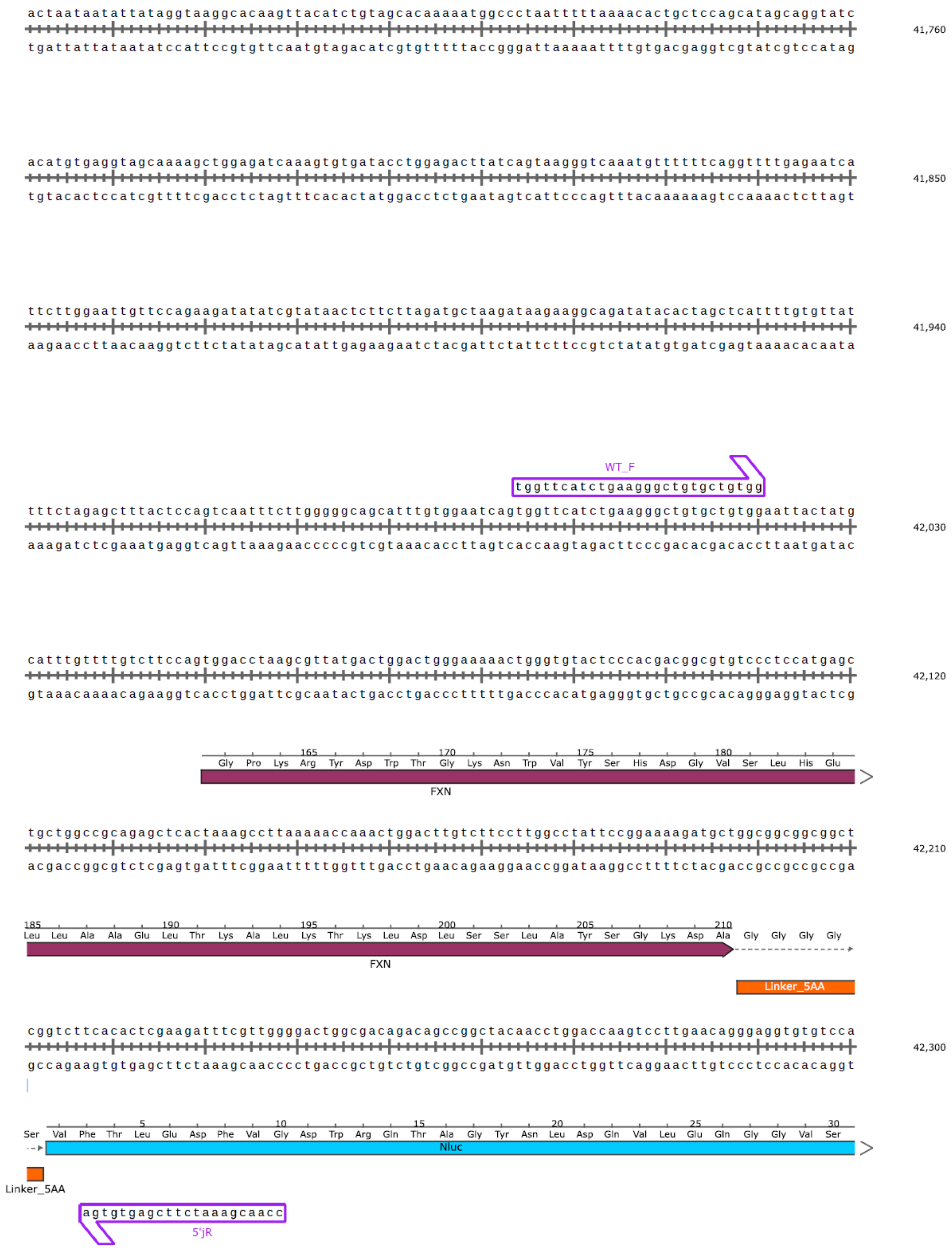
actaataatattataggtaaggcacaagttacatctgtagcacaaaaatggccctaattttaaaacactgctccagcatagcaggtatc
tgattattataatatccattccgtgttcaatgtagacatcgtgtttttaccgggattaaaaatttgtgacgaggtcgtatcgtccatag
41,760
acatgtgaggtagcaaaagctggagatcaaagtgtgatacctggagacttatcagtaagggtcaaatgtttttcaggttttgagaatca
tgtacactccatcgttttcgacctctagtttcacactatggacctctgaatagtcattcccagtttacaaaaagtccaaaactcttagt
41,850
ttcttggaattgttccagaagatatatcgtataactcttcttagatgctaagataagaaggcagatatacactagctcattttgtgttat
aagaaccttaacaaggtcttctatatagcatattgagaagaatctacgattctattcttccgtctatatgtgatcgagtaaaacacaata
41,940
WT_F
tggttcatctgaagggctgtgctgtgg
tttctagagctttactccagtcaatttcttgggggcagcatttgtggaatcagtggttcatctgaagggctgtgctgtggaattactatg
aaagatctcgaaatgaggtcagttaaagaacccccgtcgtaaacaccttagtcaccaagtagacttcccgacacgacaccttaatgatac
42,030
catttgttttgtcttccagtggacctaagcgttatgactggactgggaaaaactgggtgtactcccacgacggcgtgtccctccatgagc
gtaaacaaaacagaaggtcacctggattcgcaatactgacctgaccctttttgacccacatgagggtgctgccgcacagggaggtactcg
42,120
165 170 175 180
Gly Pro Lys Arg Tyr Asp Trp Thr Gly Lys Asn Trp Val Tyr Ser His Asp Gly Val Ser Leu His Glu
FXN
tgctggccgcagagctcactaaagccttaaaaaccaaactggacttgtcttccttggcctattccggaaaagatgctggcggcggcggct
acgaccggcgtctcgagtgatttcggaattttggtttgacctgaacagaaggaaccggataaggccttttctacgaccgccgccgccga
42,210
185 190 195 200 205 210
Leu Leu Ala Ala Glu Leu Thr Lys Ala Leu Lys Thr Lys Leu Asp Leu Ser Ser Leu Ala Tyr Ser Gly Lys Asp Ala Gly Gly Gly Gly
FXN
Linker_5AA
cggtcttcacactcgaagatttcgttggggactggcgacagacagccggctacaacctggaccaagtccttgaacagggaggtgtgtcca
gccagaagtgtgagcttctaaagcaacccctgaccgctgtctgtcggccgatgttggacctggttcaggaacttgtccctccacacaggt
42,300
5 10 15 20 25 30
Ser Val Phe Thr Leu Glu Asp Phe Val Gly Asp Trp Arg Gln Thr Ala Gly Tyr Asn Leu Asp Gln Val Leu Glu Gln Gly Gly Val Ser
Nluc
Linker_5AA
agtgtgagcttctaaagcaacc
5'jR

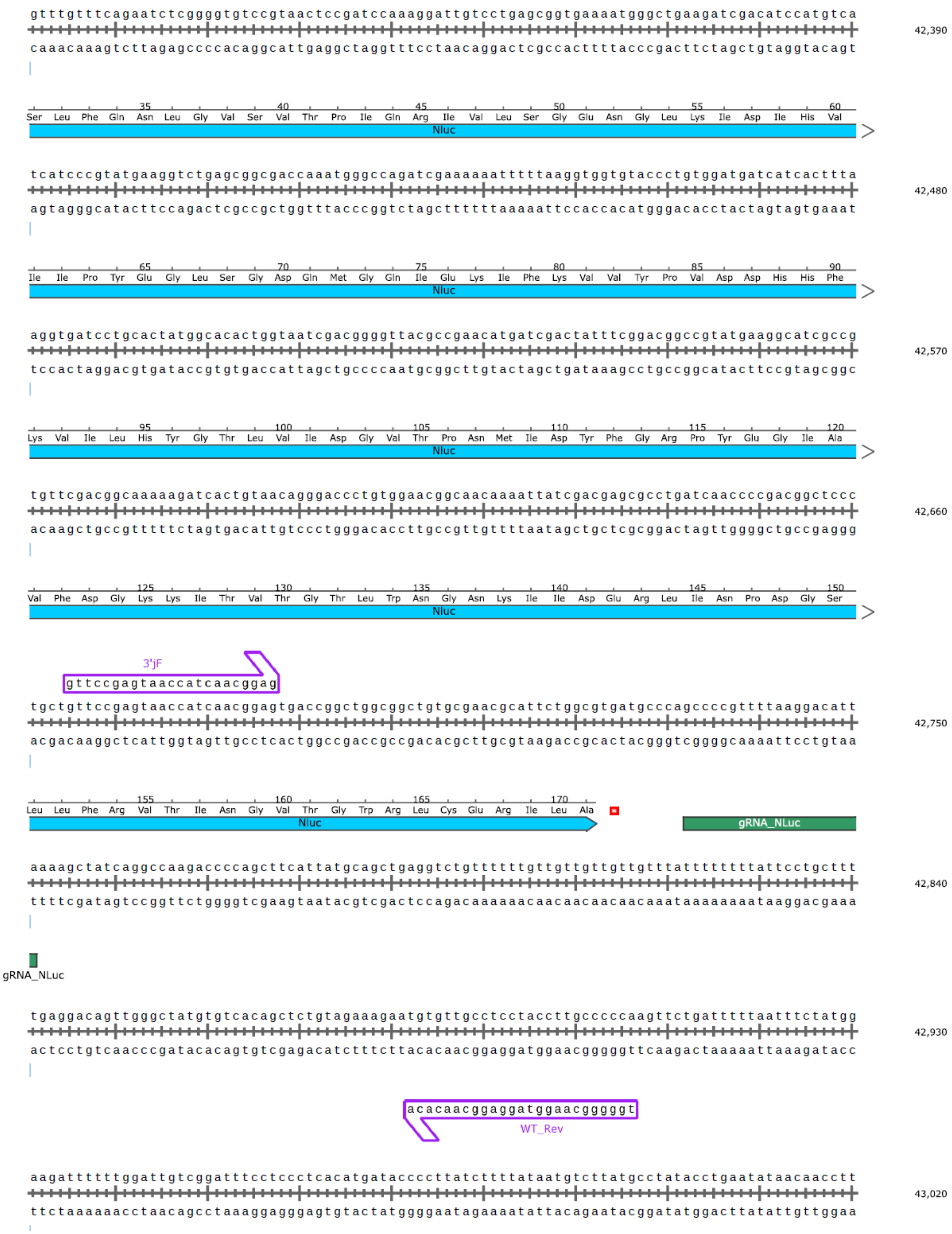
42,390
Ser Leu Phe Gln Asn Leu Gly Val Ser Val Thr Pro Ile Gln Arg Ile Val Leu Ser Gly Glu Asn Gly Leu Lys Ile Asp Ile His Val
Nluc
42,480
Ile Ile Pro Tyr Glu Gly Leu Ser Gly Asp Gln Met Gly Gln Ile Glu Lys Ile Phe Lys Val Val Tyr Pro Val Asp Asp His His Phe
Nluc
42,570
Lys Val Ile Leu His Tyr Gly Thr Leu Val Ile Asp Gly Val Thr Pro Asn Met Ile Asp Tyr Phe Gly Arg Pro Tyr Glu Gly Ile Ala
Nluc
42,660
Val Phe Asp Gly Lys Lys Ile Thr Val Thr Gly Thr Leu Trp Asn Gly Asn Lys Ile Ile Asp Glu Arg Leu Ile Asn Pro Asp Gly Ser
Nluc
3'jF
gttccgagtaaccatcaacggag
42,750
Leu Leu Phe Arg Val Thr Ile Asn Gly Val Thr Gly Trp Arg Leu Cys Glu Arg Ile Leu Ala
Nluc
gRNA_NLuc
42,840
gRNA_NLuc
42,930
acacaacggaggatggaacggggt
WT_Rev
43,020

```
taaaaaagcaaaataataagaaggaaaaattccaggagggaaaatgaattgtcttcactcttcattctttgaaggatttactgcaagaag
                                                                                            43,110
attttttcgttttattattcttccttttaaggtcctccctttacttaacagaagtgagaagtaagaaacttcctaaatgacgttcttc

tacatgaagagcagctggtcaacctgctcactgttctatctccaaatgagacacattaaagggtagcctacaaatgttttcaggcttctt
                                                                                            43,200
atgtacttctcgtcgaccagttggacgagtgacaagatagaggtttactctgtgtaatttcccatcggatgtttacaaaagtccgaagaa

tcaaagtgtaagcacttctgagctctttagcattgaagtgtcgaaagcaactcacacgggaagatcatttcttatttgtgctctgtgact
                                                                                            43,290
agtttcacattcgtgaagactcgagaaatcgtaacttcacagctttcgttgagtgtgcccttctagtaaagaataaacacgagacactga

gccaaggtgtggcctgcactgggttgtccagggagacctagtgctgtttctcccacatattcacatacgtgtctgtgtgtatatatattt
                                                                                            43,380
cggttccacaccggacgtgacccaacaggtccctctggatcacgacaaagagggtgtataagtgtatgcacagacacacatatatataaa

tttcaatttaaaggttagtatggaatcagctgctacaagaatgcaaaaaatcttccaaagacaagaaaagaggaaaaaaagccgttttca
                                                                                            43,470
aaagttaaatttccaatcataccttagtcgacgatgttcttacgttttttagaaggtttctgttcttttctcctttttttcggcaaaagt

tgagctgagtgatgtagcgtaacaaacaaaatcatggagctgaggaggtgccttgtaaacatgaaggggcagataaaggaaggagatact
                                                                                            43,560
actcgactcactacatcgcattgtttgttttagtacctcgactcctccacggaacatttgtacttccccgtctatttccttcctctatga

catgttgataaagagagccctggtcctagacatagttcagccacaaagtagttgtccctttgtggacaagtttcccaaattccctggacc
                                                                                            43,650
gtacaactatttctctcgggaccaggatctgtatcaagtcggtgtttcatcaacagggaaacacctgttcaaagggtttaagggacctgg

tctgcttccccatctgttaaatgagagaatagagtatggttgattcccagcattcagtggtcctgtcaagcaacctaacaggctagttct
                                                                                            43,740
agacgaaggggtagacaatttactctcttatctcataccaactaagggtcgtaagtcaccaggacagttcgttggattgtccgatcaaga

aattccctattgggtagatgaggggatgacaaagaacagttttaagctatataggaaacattgttattggtgttgccctatcgtgattt
                                                                                            43,830
ttaagggataacccatctactcccctactgtttcttgtcaaaattcgatatatcctttgtaacaataaccacaacgggatagcactaaa

taagggataacccatctactccc
3'jR

cagttgaattcatgtgaaaataatagccatccttggcctggcgcggtggctcacacctgtaatcccagcacttttggaggccaaggtggg
                                                                                            43,920
gtcaacttaagtacacttttattatcggtaggaaccggaccgcgccaccgagtgtggacattagggtcgtgaaaacctccggttccaccc

tggatcacctgaggtcaggagttcaagaccagcctggccaacatgatgaaaccccgtctctactaaaaatacaaaaaatt    3'
                                                                                ••• 44,000
acctagtggactccagtcctcaagttctggtcggaccggttgtactactttggggcagagatgatttttatgtttttaa    5'
```

Supplementary Figure 2: Screening of an epigenetic compound library to identify novel inducers of *FXN* expression. Complete datasets from two rounds of screening, four 96-well plates each, are presented. All compounds of the APExBIO DiscoveryProbe™ Epigenetics Compound Library were tested at 10 μM concentration for 24 h. Luminescence signal was plotted independently per each plate. Green line corresponds to plate average signal; dashed lines indicate average signal plus 1, 2 and 3 standard deviations of the mean.

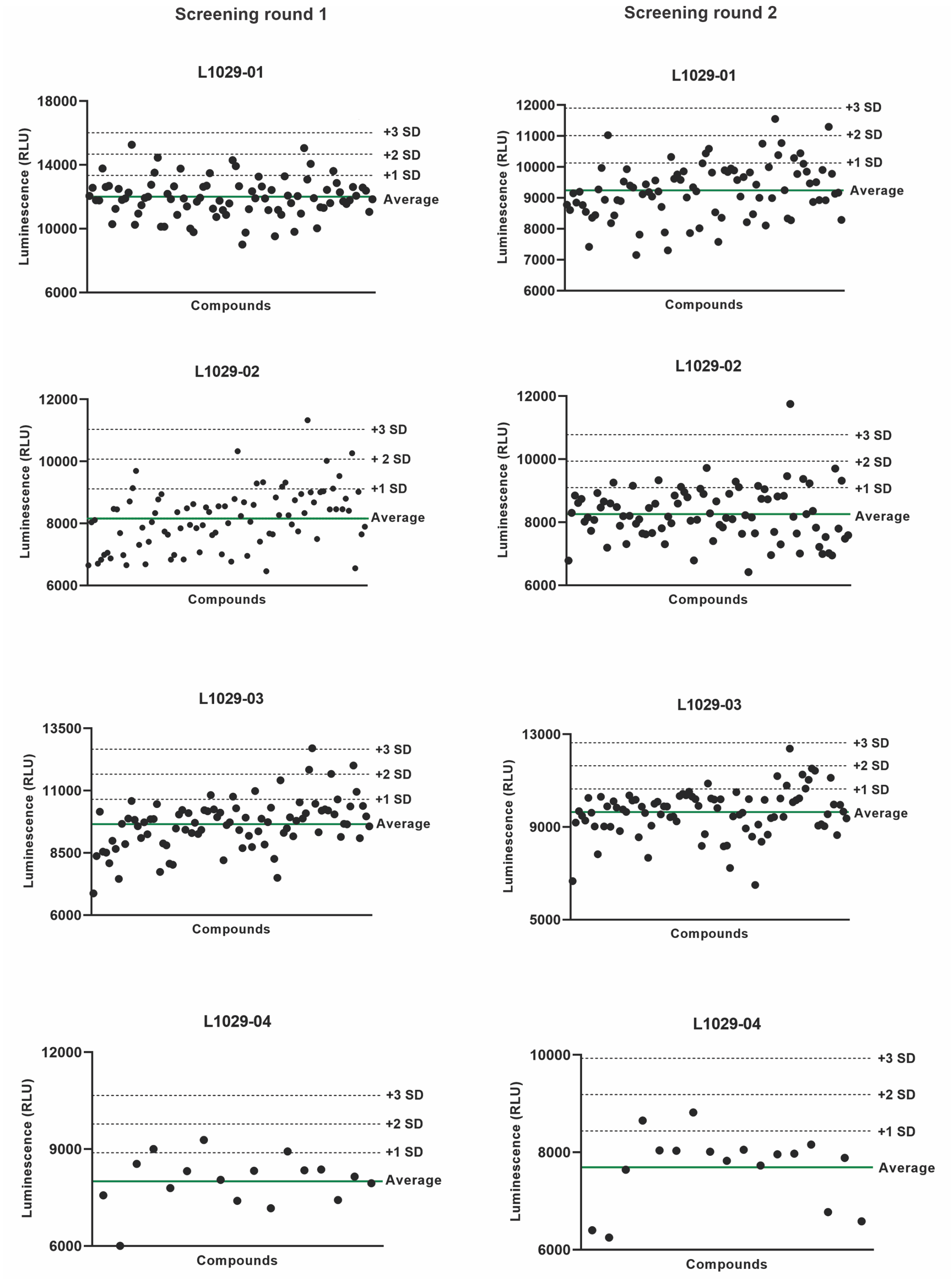

Supplementary Figure 3: Effect of selected compounds on *FXN* expression in control NPCs. (A) Results of qRT-PCR analysis of *FXN* mRNA expression upon 48 h treatment with 10 µM CI994, Mocetinostat, Entinostat, UF 010 and Cerdulatinib. Results represent mean ± SD from three technical repeats; * indicates $p<0.05$ (one-way ANOVA). DMSO treatment served as a control. (B) FXN protein expression determined by western blot after treatment with indicated compounds for 48 h at 10 µM. Signals corresponding to mature FXN (FXN-M) and intermediate FXN (FXN-I) are indicated. GAPDH and Ponceau S (C) staining served as loading controls. (D) Quantification of FXN-M levels shown in panel B relative to Ponceau S staining.

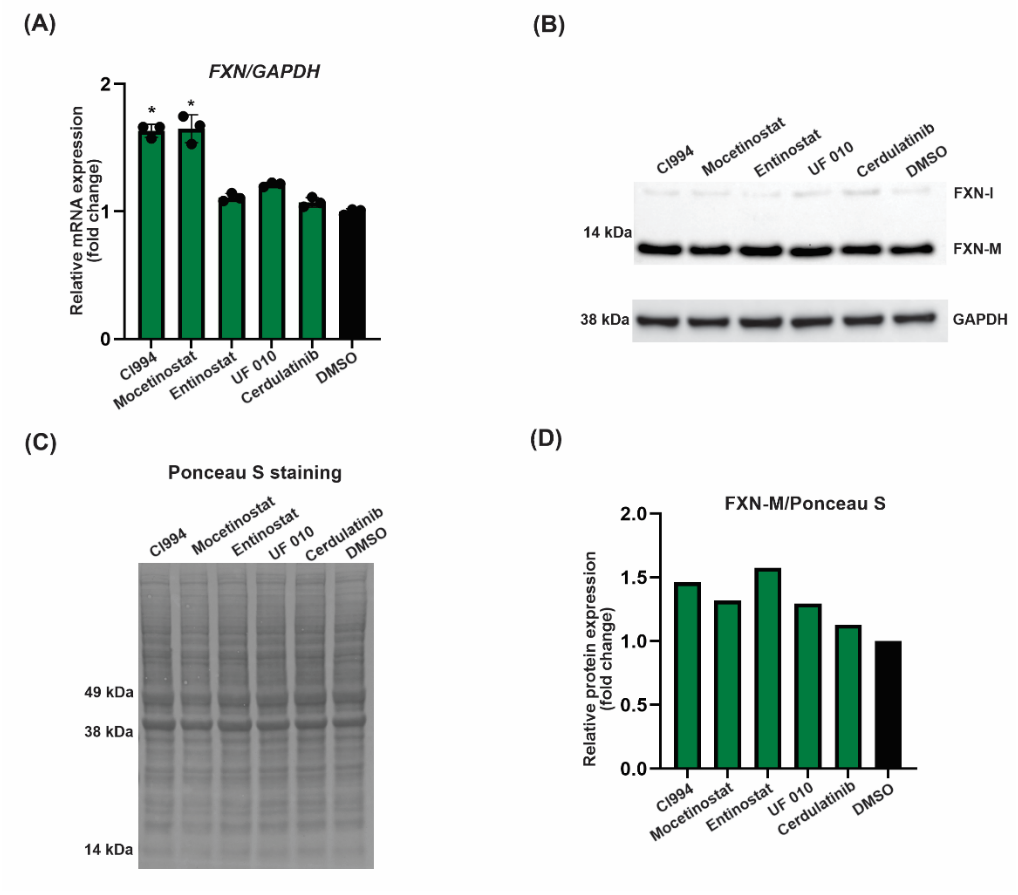

Supplementary Table 1: Characteristics of induced pluripotent stem cell lines used in the study.

Type:	Age of disease onset:	Sex:	GAA repeats:
FRDA 4676	10	M	867/867
FRDA 86	18	F	450/450
FRDA 4259	15	M	550/830
Control 7522	-	F	-

Supplementary Table 2: Complete list of compounds included in APExBIO DiscoveryProbe™ Epigenetics Compound Library. For each compound the CAS number, molecular weight, formula, targeted pathway and molecular target are presented.

Item Name	CAS Number	M.w.	Formula	Pathway	Target	Information
Daptomycin	103060-53-3	1620.67	C72H101N17O26	Cancer Biology	DNA Synthesis	Calcium-dependent antibiotic
Gemcitabine HCl	122111-03-9	299.66	C9H11F2N3O4.HCl	Cancer Biology	DNA Synthesis	Inhibits DNA synthesis,deoxycytidine analog
XAV-939	284028-89-3	312.31	C14H11F3N2OS	Cancer Biology	PARP	Tankyrase 1/2 inhibitor
Decitabine (NSC127716, 5AZA-CdR)	2353-33-5	228.08	C8H12N4O4	Cancer Biology	DNA Methyltransferase	Deoxycytidine analog and cellular differentiation inducer
5-Azacytidine	320-67-2	244.2	C8H12N4O5	Cancer Biology	DNA Methyltransferase	DNA methyltransferase inhibitor.
BIX 01294	935693-62-2	490.65	C28H38N6O2	Cancer Biology	Histone Methyltransferase	G9a and GLP inhibitor
Bromodomain Inhibitor, (+)-JQ1	1268524-70-4	456.99	C23H25ClN4O2S	Cancer Biology	Bromodomain	BET bromodomain inhibitor
Lomeguatrib	192441-08-0	326.17	C10H8BrN5OS	Cancer Biology	DNA Methyltransferase	MGMT inhibitor
RG 108	48208-26-0	334.33	C19H14N2O4	Cancer Biology	DNA Methyltransferase	DNA methyltransferase inhibitor
UNC0638	1255580-76-7	509.72	C30H47N5O2	Cancer Biology	Histone Methyltransferase	G9a/GLP HMTase inhibitor, potent and selective
Zebularine	3690-10-6	228.2	C9H12N2O5	Cancer Biology	DNA Methyltransferase	DNA methylation inhibitor
Carboplatin	41575-94-4	371.25	C6H12N2O4Pt	Cancer Biology	DNA Synthesis	Antitumor agent that forms platinum-DNA adducts.
Ellagic acid	476-66-4	302.19	C14H6O8	Cancer Biology	Topoisomerase	Casein kinase 2 (CK2) inhibitor
(-)-Epigallocatechin gallate (EGCG)	989-51-5	458.37	C22H18O11	Cancer Biology	PKC	Antioxidant, antiangiogenic and antitumor agent
ABT-888 (Veliparib)	912444-00-9	244.3	C13H16N4O	Cancer Biology	PARP	Potent PARP inhibitor
Ruxolitinib (INCB018424)	941678-49-5	306.37	C17H18N6	Cancer Biology	JAK	JAK inhibitor
AZD1152	722543-31-9	587.54	C26H31FN7O6P	Cancer Biology	Aurora Kinase	Aurora B kinase inhibitor,highly potent and selective
Bardoxolone methyl	218600-53-4	505.69	C32H43NO4	Cancer Biology	JAK	IKK inhibitor, potent antioxidant inflammation modulator
Curcumin	458-37-7	368.38	C21H20O6	Cancer Biology	KEAP1-Nrf2	Tyrosinase inhibitor
CX-6258	1202916-90-2	461.94	C26H24ClN3O3	Cancer Biology	Pim	Pan-Pim kinases inhibitor
Daminozide	1596-84-5	160.17	C6H12N2O3	Cancer Biology	HDAC	KDM2A inhibitor
GSK126	1346574-57-9	526.67	C31H38N6O2	Cancer Biology	EZH2	EZH2 inhibitor
GSK343	1346704-33-3	541.69	C31H39N7O2	Cancer Biology	EZH2	EZH2 inhibitor,potent,selective and cell permeable
JANEX-1	202475-60-3	297.31	C16H15N3O3	Cancer Biology	JAK	JAK3 inhibitor
MI-2	1271738-62-5	375.55	C18H25N5S2	Cancer Biology	Menin-MLL	Menin-MLL inhibitor
MK-4827	1038915-60-4	320.39	C19H20N4O	Cancer Biology	PARP	PARP-1/-2 inhibitor,potent and selective
NVP-BSK805	1092499-93-8	490.55	C27H28F2N6O	Cancer Biology	JAK	ATP-competitive JAK2 inhibitor
OTX-015	202590-98-5	491.99	C25H22ClN5O2S	Cancer Biology	Bromodomain	BRD inhibitor
Pyridone 6	457081-03-7	309.34	C18H16FN3O	Cancer Biology	JAK	Pan-JAK inhibitor
Reversine	656820-32-5	393.49	C21H27N7O	Chromatin/Epigenetics	Aurora Kinase	A3 adenosine receptor antagonist,ARK-1/-2/-3 inhibitor
RG2833	1215493-56-3	339.43	C20H25N3O2	Cancer Biology	HDAC	Brain-penetrant HDAC inhibitor
Ruxolitinib phosphate	1092939-17-7	404.36	C17H21N6O4P	Chromatin/Epigenetics	JAK	JAK1/JAK2 inhibitor
SB1317	937270-47-8	372.46	C23H24N4O	Cancer Biology	JAK	CDK,JAK and FLT inhibitor
Tasquinimod	254964-60-8	406.36	C20H17F3N2O4	Cancer Biology	HDAC	Antiangiogenic and antineoplastic agent
UNC1215	1415800-43-9	529.72	C32H43N5O2	Chromatin/Epigenetics	Bromodomain	Chemical probe for the methyllysine (Kme)
XL019	945755-56-6	444.53	C25H28N6O2	Chromatin/Epigenetics	JAK	JAK2 inhibitor,potent and selective
XL228	898280-07-4	437.54	C22H31N9O	Cell Cycle/Checkpoint	Aurora Kinase	IGF1R/AURORA /FGFR1-3/ABL/SRC family kinases inhibitor
Veliparib dihydrochloride	912445-05-7	317.21	C13H18Cl2N4O	Chromatin/Epigenetics	PARP	PARP-1/PARP-2 inhibitor
AZD1208	1204144-28-4	379.48	C21H21N3O2S	Chromatin/Epigenetics	Pim	PIM kinase inhibitor
A-769662	844499-71-4	360.39	C20H12N2O3S	Cancer Biology	Others	AMPK activator,potent and reversible
BI 2536	755038-02-9	521.67	C28H39N7O3	Cell Cycle/Checkpoint	PLK	Plk1 inhibitor,potent and ATP-competitive
Rocilinostat (ACY-1215)	1316214-52-4	433.5	C24H27N5O3	DNA Damage/DNA Repair	HDAC	Selective HDAC6 inhibitor
Vorinostat (SAHA, MK0683)	149647-78-9	264.3	C14H20N2O3	DNA Damage/DNA Repair	HDAC	HDAC inhibitor
Mocetinostat (MGCD0103, MG0103)	726169-73-9	396.44	C23H20N6O	DNA Damage/DNA Repair	HDAC	HDAC inhibitor,isotype-selective and potent
JNJ-26481585	875320-29-9	394.48	C21H26N6O2	Cancer Biology	HDAC	Potent HDAC inhibitor
PCI-34051	950762-95-5	296.32	C17H16N2O3	DNA Damage/DNA Repair	HDAC	HDAC8 inhibitor,potent and selective
CUDC-101	1012054-59-9	434.49	C24H26N4O4	DNA Damage/DNA Repair	HDAC	Multitargeted HDAC inhibitor
ITF2357 (Givinostat)	732302-99-7	475.97	C24H27N3O4.HCl.H2O	DNA Damage/DNA Repair	HDAC	HDAC inhibitor
MC1568	852475-26-4	314.31	C17H15FN2O3	Cancer Biology	HDAC	Class II HDAC inhibitor,potent and selective
Pracinostat (SB939)	929016-96-6	358.48	C20H30N4O2	DNA Damage/DNA Repair	HDAC	Pan-HDAC inhibitor
Belinostat (PXD101)	414864-00-9	318.35	C15H14N2O4S	DNA Damage/DNA Repair	HDAC	Hydroxamate-type HDAC inhibitor
CUDC-907	1339928-25-4	508.55	C23H24N8O4S	DNA Damage/DNA Repair	HDAC	Potent PI3K/HDAC inhibitor
PCI-24781 (CRA-024781)	783355-60-2	397.42	C21H23N3O5	Cancer Biology	HDAC	Pan-HDAC inhibitor
Valproic acid sodium salt (Sodium valproate)	1069-66-5	166.19	C8H15NaO2	DNA Damage/DNA Repair	HDAC	HDAC inhibitor
Droxinostat	99873-43-5	243.69	C11H14ClNO3	DNA Damage/DNA Repair	HDAC	Selective HDAC inhibitor
Tubastatin A	1252003-15-8	335.4	C20H21N3O2	DNA Damage/DNA Repair	HDAC	HDAC6 inhibitor,potent and selective
CI994 (Tacedinaline)	112522-64-2	269.3	C15H15N3O2	Cancer Biology	HDAC	HDAC inhibitor
LAQ824 (NVP-LAQ824,Dacinostat)	404951-53-7	379.46	C22H25N3O3	DNA Damage/DNA Repair	HDAC	HDAC inhibitor,potent and novel
AR-42 (OSU-HDAC42)	935881-37-1	312.36	C18H20N2O3	DNA Damage/DNA Repair	HDAC	HDAC inhibitor,novel and potent
M344	251456-60-7	307.39	C16H25N3O3	DNA Damage/DNA Repair	HDAC	HDAC inhibitor,potent and cell-permeable
Scriptaid	287383-59-9	326.35	C18H18N2O4	Cancer Biology	HDAC	HDAC inhibitor,novel and cell-permeable
Sodium Phenylbutyrate	1716-12-7	186.18	C10H11O2.Na	DNA Damage/DNA Repair	HDAC	Histone deacetylase inhibitor
MLN8237 (Alisertib)	1028486-01-2	518.92	C27H20ClFN4O4	Chromatin/Epigenetics	Aurora Kinase	Aurora A Kinase inhibitor, Potent and selective
VX-680 (MK-0457,Tozasertib)	639089-54-6	464.59	C23H28N8OS	Cancer Biology	Aurora Kinase	Aurora kinase inhibitor
Barasertib (AZD1152-HQPA)	722544-51-6	507.56	C26H30FN7O3	Chromatin/Epigenetics	Aurora Kinase	Aurora Kinase B inhibitor, Potent and selective
ZM 447439	331771-20-1	513.59	C29H31N5O4	Chromatin/Epigenetics	Aurora Kinase	Aurora Kinase inhibitor,potent and selective
MLN8054	869363-13-3	476.86	C25H15ClF2N4O2	Chromatin/Epigenetics	Aurora Kinase	Aurora A inhibitor
JNJ-7706621	443797-96-4	394.36	C15H12F2N6O3S	Cancer Biology	Aurora Kinase	Potent CDK/Aurora kinase inhibitor
Danusertib (PHA-739358)	827318-97-8	474.55	C26H30N6O3	Tyrosine Kinase	c-RET	Pan-aurora kinase inhibitor
AT9283	896466-04-9	381.43	C19H23N7O2	Chromatin/Epigenetics	Aurora Kinase	Aurora kinase/JAK inhibitor
Hesperadin	422513-13-1	516.65	C29H32N4O3S	Chromatin/Epigenetics	Aurora Kinase	Aurora B kinase inhibitor
AMG-900	945595-80-2	503.58	C28H21N7OS	Cancer Biology	Aurora Kinase	Aurora kinase inhibitor
MK-5108 (VX-689)	1010085-13-8	461.94	C22H21ClFN3O3S	Chromatin/Epigenetics	Aurora Kinase	Aurora-A kinase inhibitor,highly selective
SNS-314 Mesylate	1146618-41-8	527.04	C18H15ClN6OS2.CH4O3S	Chromatin/Epigenetics	Aurora Kinase	Aurora A/B/C kinases inhibitor, potent and selective
PHA-680632	398493-79-3	501.62	C28H35N7O2	Chromatin/Epigenetics	Aurora Kinase	Aurora kinase inhibitor,novel and potent
KW 2449	1000669-72-6	332.4	C20H20N4O	Cancer Biology	FLT3	Multikinase inhibitor
TAK-901	934541-31-8	504.64	C28H32N4O3S	Chromatin/Epigenetics	Aurora Kinase	Novel Aurora A/B inhibitor
CYC116	693228-63-6	368.46	C18H20N6OS	Chromatin/Epigenetics	Aurora Kinase	Potent Aurora A/B inhibitor
Aurora A Inhibitor I	1158838-45-9	588.07	C31H31ClFN7O2	Cell Cycle/Checkpoint	Aurora Kinase	Aurora A inhibitor
ENMD-2076 L-(+)-Tartaric acid	1291074-87-7	525.56	C25H31N7O6	Chromatin/Epigenetics	Aurora Kinase	Aurora kinases inhibitor
ENMD-2076	934353-76-1	375.47	C21H25N7	Chromatin/Epigenetics	Aurora Kinase	Selective Aurora A/Flt3 inhibitor
CCT129202	942947-93-5	497.02	C23H25ClN8OS	Cancer Biology	Aurora Kinase	Aurora kinase inhibitor,ATP-competitive and potent
CCT137690	1095382-05-0	551.48	C26H31BrN8O	Chromatin/Epigenetics	Aurora Kinase	Aurora A/B/C inhibitor
Tofacitinib (CP-690550) Citrate	540737-29-9	504.49	C16H20N6O.C6H8O7	Chromatin/Epigenetics	JAK	Potent JAK inhibitor
TG101348 (SAR302503)	936091-26-8	524.68	C27H36N6O3S	Chromatin/Epigenetics	JAK	JAK-2 inhibitor,potent and selective
AZD1480	935666-88-9	348.77	C14H14ClFN8	Cancer Biology	JAK	JAK2 inhibitor,ATP-competitive and novel
Tofacitinib (CP-690550,Tasocitinib)	477600-75-2	312.37	C16H20N6O	Chromatin/Epigenetics	JAK	Janus kinase inhibitor
AG-490	133550-30-8	294.3	C17H14N2O3	JAK/STAT Signaling	EGFR	JAK2/EGFR inhibitor
WP1066	857064-38-1	356.22	C17H14BrN3O	Chromatin/Epigenetics	JAK	JAK2/STAT3 inhibitor,cell-permeable
Baricitinib (LY3009104, INCB028050)	1187594-09-7	371.42	C16H17N7O2S	Cancer Biology	JAK	JAK1/JAK2 inhibitor,selective orally bioavailable
CYT387	1056634-68-4	414.46	C23H22N6O2	Chromatin/Epigenetics	JAK	JAK-1/-2 inhibitor,ATP competitive
TG101209	936091-14-4	509.67	C26H35N7O2S	Tyrosine Kinase	c-RET	JAK2/3 inhibitor
CEP-33779	1257704-57-6	462.57	C24H26N6O2S	Cancer Biology	JAK	JAK2 inhibitor,highly selective
LY2784544	1229236-86-5	469.94	C23H25ClFN7O	Chromatin/Epigenetics	JAK	JAK2 inhibitor,highly potent and selective
WHI-P154	211555-04-3	376.2	C16H14BrN3O3	Cancer Biology	JAK	JAK3 inhibitor
ZM 39923 HCl	1021868-92-7	367.91	C23H25NO.HCl	JAK/STAT Signaling	JAK	JAK3 inhibitor,potent and selective
BMS-911543	1271022-90-2	432.52	C23H28N8O	JAK/STAT Signaling	JAK	JAK2 inhibitor,selective small molecule
BMN 673	1207456-01-6	380.35	C19H14F2N6O	DNA Damage/DNA Repair	PARP	Potent PARP inhibitor
Olaparib (AZD2281, Ku-0059436)	763113-22-0	434.46	C24H23FN4O3	Cancer Biology	PARP	Potent PARP1/PARP2 inhibitor
Rucaparib (AG-014699,PF-01367338)	459868-92-9	421.36	C19H18FN3O.H3PO4	Chromatin/Epigenetics	PARP	Potent PARP inhibitor
Iniparib (BSI-201)	160003-66-7	292.03	C7H5IN2O3	Chromatin/Epigenetics	PARP	PARP1 inhibitor,intravenously adminsitered
AG-14361	328543-09-5	320.39	C19H20N4O	DNA Damage/DNA Repair	PARP	Potent PARP1 inhibitor
PJ34 hydrochloride	344458-15-7	331.8	C17H17N3O2.HCl	Cancer Biology	PARP	PARP inhibitor,potent and cell-permeable
A-966492	934162-61-5	324.35	C18H17FN4O	Chromatin/Epigenetics	PARP	PARP-1/-2 inhibitor, highly potent
INO-1001	3544-24-9	136.15	C7H8N2O	Chromatin/Epigenetics	PARP	Potent PARP inhibitor
UPF 1069	1048371-03-4	279.29	C17H13NO3	Chromatin/Epigenetics	PARP	Selective PARP2 inhibitor
AZD2461	1174043-16-3	395.43	C22H22FN3O3	Cancer Biology	PARP	Novel PARP inhibitor
EPZ5676	1380288-87-8	562.71	C30H42N8O3	Chromatin/Epigenetics	Histone Methyltransferase	DOT1L inhibitor,potent and SAM competitive
SGC 0946	1561178-17-3	618.57	C28H40BrN7O4	Chromatin/Epigenetics	Histone Methyltransferase	DOT1L inhibitor,highly potent and selective
Entacapone	130929-57-6	305.29	C14H15N3O5	Chromatin/Epigenetics	Histone Methyltransferase	COMT inhibitor
EPZ004777	1338466-77-5	539.67	C28H41N7O4	Cancer Biology	Histone Methyltransferase	DOT1L inhibitor
EPZ005687	1396772-26-1	539.67	C32H37N5O3	Chromatin/Epigenetics	Histone Methyltransferase	EZH2 inhibitor,potent and selective
Thioguanine	154-42-7	167.19	C5H5N5S	DNA Damage/DNA Repair	DNA Methyltransferase	Purine antimetabolite
SRT1720 HCl	1001645-58-4	506.02	C25H23N7OS.HCl	Chromatin/Epigenetics	Sirtuin	SIRT1 activator
EX 527 (SEN0014196)	49843-98-3	248.71	C13H13ClN2O	Cancer Biology	Sirtuin	SIRT1 inhibitor
Resveratrol	501-36-0	228.24	C14H12O3	Chromatin/Epigenetics	Sirtuin	SIRT1 activator
Sirtinol	410536-97-9	394.47	C26H22N2O2	Chromatin/Epigenetics	Sirtuin	SIRT inhibitor
PFI-1 (PF-6405761)	1403764-72-6	347.39	C16H17N3O4S	Chromatin/Epigenetics	Bromodomain	BET inhibitor
Bromosporine	1619994-69-2	404.44	C17H20N6O4S	Cancer Biology	Bromodomain	Bromodomain inhibitor,non-selective
FG-4592 (ASP1517)	808118-40-3	352.34	C19H16N2O5	Angiogenesis	HIF	HIF prolyl-hydroxylase inhibitor
2-Methoxyestradiol (2-MeOE2)	362-07-2	302.41	C19H26O3	Apoptosis	Apoptosis Inducers	Apoptotic, antiproliferative and antiangiogenic agent
IOX2(Glycine)	931398-72-0	352.34	C19H16N2O5	Angiogenesis	HIF	HIF-1α prolyl hydroxylase-2 (PHD2) inhibitor
GSK J4 HCl	1373423-53-0	453.96	C24H27N5O2.HCl	Cancer Biology	Histone Demethylases	Inhibitor of H3K27 demethylase JMJD3,potent and cell-permeable
GSK J1	1373422-53-7	389.45	C22H23N5O2	Chromatin/Epigenetics	Histone Demethylases	H3K27 demethylase JMJD3 inhibitor
SGI-1776 free base	1025065-69-3	405.42	C20H22F3N5O	Chromatin/Epigenetics	Pim	Pim kinase inhibitor,ATP-competitive
SMI-4a	438190-29-5	273.23	C11H6F3NO2S	JAK/STAT Signaling	Pim	Potent Pim inhibitor
Tenovin-1	380315-80-0	369.5	C20H23N3O2S	Apoptosis	p53	SIRT2 inhibitor, activates p53
Mitomycin C	50-07-7	334.33	C15H18N4O5	Apoptosis	Apoptosis Inducers	Inhibits DNA synthesis,antibiotic and antitumor agent
Anacardic acid	16611-84-0	348.52	C22H36O3	Chromatin/Epigenetics	Aurora Kinase	HAT inhibitor
SGC-CBP30	1613695-14-9	509.04	C28H33ClN4O3	Cancer Biology	Bromodomain	Inhibitor of CREBBP/EP300 bromodomain,potent
KD 5170	940943-37-3	451.56	C20H25N3O5S2	DNA Damage/DNA Repair	HDAC	HDAC inhibitor
LMK 235	1418033-25-6	294.35	C15H22N2O4	DNA Damage/DNA Repair	HDAC	HDAC4/HDAC5 inhibitor
NCH 51	848354-66-5	390.56	C20H26N2O2S2	Cancer Biology	HDAC	Histone deacetylase (HDAC) inhibitor
NSC 3852	3565-26-2	174.16	C9H6N2O2	DNA Damage/DNA Repair	HDAC	HDAC inhibitor
SBHA	38937-66-5	204.22	C8H16N2O4	DNA Damage/DNA Repair	HDAC	HDAC1/HDAC3 inhibitor,cell-permeable
TC-H 106	937039-45-7	339.43	C20H25N3O2	Cancer Biology	HDAC	HDAC inhibitor
UNC 0224	1197196-48-7	485.67	C26H43N7O2	Cancer Biology	Histone Methyltransferase	G9a histone methyltransferase inhibitor
UNC 0646	1320288-17-2	621.9	C36H59N7O2	Chromatin/Epigenetics	Histone Methyltransferase	G9a and GLP inhibitor

DMOG	89464-63-1	175.14	C6H9NO5	Chromatin/Epigenetics	HIF	Competitive HIF-PH inhibitor, cell-permeable
KC7F2	927822-86-4	570.38	C16H16Cl4N2O4S4	Cancer Biology	HIF	HIF-1α inhibitor
PX 12	141400-58-0	188.31	C7H12N2S2	Chromatin/Epigenetics	HIF	Trx-1 inhibitor
1,2,3,4,5,6-Hexabromocyclohexane	1837-91-8	557.54	C6H6Br6	Chromatin/Epigenetics	JAK	JAK2 tyrosine kinase inhibitor
ZM 449829	4452-6-6	182.22	C13H10O	Chromatin/Epigenetics	JAK	JAK3 inhibitor
TCS PIM-1 1	491871-58-0	367.2	C18H11BrN2O2	Chromatin/Epigenetics	Pim	Pim-1 kinase inhibitor,ATP-competitve
4-HQN	491-36-1	146.15	C8H6N2O	Chromatin/Epigenetics	PARP	PARP inhibitor
JW 55	664993-53-7	434.48	C25H26N2O5	Cancer Biology	PARP	PARP domain inhibitor
WIKI4	838818-26-1	521.59	C29H23N5O3S	Chromatin/Epigenetics	PARP	Inhibitor of Wnt/ß-Catenin signaling and tankyrase
Sal 003	1164470-53-4	463.21	C18H15Cl4N3OS	Cancer Biology	Protein Ser/Thr Phosphatases	Cellular phosphatase complex inhibitor
NSC 87877	56990-57-9	459.45	C19H13N3O7S2	Chromatin/Epigenetics	Protein Ser/Thr Phosphatases	Shp2 /shp1 PTP inhibitor
Ofloxacin	82419-36-1	361.37	C18H20FN3O4	DNA Damage/DNA Repair	Topoisomerase	Fluoroquinolones,antibiotics
Fasudil (HA-1077) HCl	105628-07-7	327.83	C14H17N3O2S.HCl	TGF-β / Smad Signaling	ROCK	Protein kinase inhibitor
Entinostat (MS-275,SNDX-275)	209783-80-2	376.4	C21H20N4O3	DNA Damage/DNA Repair	HDAC	HDAC1 and HDAC3 inhibitor
Romidepsin (FK228, depsipeptide)	128517-07-7	540.7	C24H36N4O6S2	DNA Damage/DNA Repair	HDAC	HDAC1/HDAC2 inhibitor,potent and selective
Panobinostat (LBH589)	404950-80-7	349.43	C21H23N3O2	DNA Damage/DNA Repair	HDAC	HDAC inhibitor
(-)-JQ1	1268524-71-5	456.99	C23H25ClN4O2S	Chromatin/Epigenetics	Bromodomain	BET bromodomain inhibitor
3-Deazaneplanocin A (DZNep) hydrochloride	120964-45-6	298.73	C12H14N4O3.HCl	Stem Cell	EZH2	SAHH and ENZ2 inhibitor
Trichostatin A (TSA)	58880-19-6	302.37	C17H22N2O3	DNA Damage/DNA Repair	HDAC	HDAC inhibitor
Nanaomycin A	52934-83-5	302.28	C16H14O6	Chromatin/Epigenetics	DNA Methyltransferase	DNMT3B inhibitor
EPZ-6438	1403254-99-8	572.74	C34H44N4O4	Chromatin/Epigenetics	Histone Methyltransferase	EZH2 inhibitor,potent and selective
CID 2011756	638156-11-3	396.87	C22H21ClN2O3	Chromatin/Epigenetics	Protein Ser/Thr Phosphatases	PKD inhibitor
TAK-632	1228591-30-7	554.52	C27H18F4N4O3S	MAPK Signaling	Raf	Pan-RAF inhibitor,potent and selective
Splitomicin	5690-3-9	198.22	C13H10O2	Chromatin/Epigenetics	Sirtuin	inhibitor of Sir2p and fMLP-induced free radicals
Tenovin-6	1011557-82-6	454.6	C25H34N4O2S	Chromatin/Epigenetics	Sirtuin	SIRT inhibitor and p53 activator
Go 6983	133053-19-7	442.51	C26H26N4O3	TGF-β / Smad Signaling	PKC	pan-PKC inhibitor
Cytarabine	147-94-4	243.2	C9H13N3O5	DNA Damage/DNA Repair	DNA Synthesis	cytotoxic agent, blocks DNA synthesis
Sotrastaurin (AEB071)	425637-18-9	438.48	C25H22N6O2	TGF-β / Smad Signaling	PKC	PKC inhibitor
Tubastatin A HCl	1310693-92-5	371.86	C20H21N3O2.HCl	DNA Damage/DNA Repair	HDAC	HDAC6 inhibitor,potent and selective
Tankyrase Inhibitors (TNKS) 49		448.54	C24H24N4O3S	Chromatin/Epigenetics	PARP	Tankyrase inhibitor
Sephin1	13098-73-2	196.64	C8H9ClN4	Chromatin/Epigenetics	Protein Ser/Thr Phosphatases	Selective PPP1R15A inhibitor
MCB-613	1162656-22-5	305.37	C19H19N3O	Chromatin/Epigenetics	Histone Acetyltransferases	stimulator of steroid receptor coactivator (SRC)
RGFP966	1357389-11-7	362.4	C21H19FN4O	DNA Damage/DNA Repair	HDAC	Specific HDAC3 inhibitor
TMP269	1314890-29-3	514.52	C25H21F3N4O3S	DNA Damage/DNA Repair	HDAC	HDAC 4/5/7/9 inhibitor
MK-8745	885325-71-3	431.91	C20H19ClFN5OS	Cell Cycle/Checkpoint	Aurora Kinase	Aurora A inhibitor,potent and selective
UNC669	1314241-44-5	338.24	C15H20BrN3O	Chromatin/Epigenetics	Bromodomain	L3MBTL antagonist,potent and selective
HTH-01-015	1613724-42-7	468.55	C26H28N8O	PI3K/Akt/mTOR Signaling	AMPK	NUAK1 inhibitor,highly specific and selective
Rucaparib (free base)	283173-50-2	323.36	C19H18FN3O	Chromatin/Epigenetics	PARP	Potent PARP inhibitor
MS436	1395084-25-9	383.42	C18H17N5O3S	Chromatin/Epigenetics	Bromodomain	BRD4 inhibitor
GSK1324726A	1300031-52-0	434.91	C25H23ClN2O3	Chromatin/Epigenetics	Bromodomain	BET proteins inhibitor
EI1	1418308-27-6	390.48	C23H26N4O2	Stem Cell	EZH2	EZH2 inhibitor
CPI-203	1446144-04-2	399.9	C19H18ClN5OS	Chromatin/Epigenetics	Bromodomain	BET bromodomain inhibitor
UNC 0631	1320288-19-4	635.93	C37H61N7O2	Chromatin/Epigenetics	Histone Methyltransferase	G9a inhibitor
GLPG0634	1206161-97-8	425.5	C21H23N5O3S	JAK/STAT Signaling	JAK	JAK1 inhibitor
ABC294640	915385-81-8	380.91	C23H25ClN2O	Chromatin/Epigenetics	Sphingosine Kinase-2	Sphingosine kinase 2 inhibitor,selective and competitive
Valproic acid	99-66-1	144.21	C8H16O2	DNA Damage/DNA Repair	HDAC	HDAC1 inhibitor
Dorsomorphin 2HCl	1219168-18-9	472.41	C24H25N5O.2HCl	PI3K/Akt/mTOR Signaling	AMPK	AMPK inhibitor
Phenformin HCl	834-28-6	241.72	C10H16ClN5	Others	Others	biguanidine drug with anti-diabetic activity
WZ4003	1214265-58-3	496.99	C25H29ClN6O3			NUAK1/2 inhibitor, potent and selective
Divalproex Sodium	76584-70-8	310.41	C8H16O2.C8H15O2.Na	Ubiquitination/ Proteasome	Autophagy	Epilepsy/migraines/bipolar disorder medicine
Procarbazine HCl	366-70-1	257.76	C12H19N3O.HCl	DNA Damage/DNA Repair	DNA Synthesis	Antineoplastic chemotherapy drug
Mizoribine	50924-49-7	259.22	C9H13N3O6	Others	IMPDH	DNA/RNA synthesis inhibitor
I-BET-762	1260907-17-2	423.9	C22H22ClN5O2	Chromatin/Epigenetics	Bromodomain	BET inhibitor,highly potent
RVX-208	1044870-39-4	370.4	C20H22N2O5	Chromatin/Epigenetics	Bromodomain	Potent BET bromodomain inhibitor
I-BET151 (GSK1210151A)	1300031-49-5	415.44	C23H21N5O3	Chromatin/Epigenetics	Bromodomain	Selective BET inhibitor
C646	328968-36-1	445.42	C24H19N3O6	Chromatin/Epigenetics	Histone Acetyltransferases	HAT p300-CBP inhibitor,cell-permeable
JIB-04	199596-05-9	308.76	C17H13ClN4			Jumonji histone demethylase inihibitor
OG-L002	1357302-64-7	225.29	C15H15NO	Chromatin/Epigenetics	Histone Demethylases	LSD1 inhibitor,potent and specific
MM-102	1417329-24-8	669.8	C35H49F2N7O4	Chromatin/Epigenetics	Histone Methyltransferase	MLL1 inhibitor,high-affinity peptidomimetic
UNC1999	1431612-23-5	569.74	C33H43N7O2	Chromatin/Epigenetics	Histone Methyltransferase	EZH2 inhibitor
Donepezil HCl	120011-70-3	415.95	C24H30ClNO3	Chromatin/Epigenetics	Histone Acetyltransferases	AChE inhibitor
SGI-1027	1020149-73-8	461.52	C27H23N7O	Chromatin/Epigenetics	Histone Methyltransferase	DNMT inhibitor
Sodium butyrate	156-54-7	110.09	C4H7NaO2			Histone deacetylase inhibitor
Metformin HCl	1115-70-4	165.62	C4H12ClN5	Others	Others	Anti-diabetic drug
Nedaplatin	95734-82-0	303.17	C2H8N2O3Pt	GPCR/G protein	Adrenergic Receptor	DNA synthesis inhibitor
Mitoxantrone HCl	70476-82-3	517.4	C22H29ClN4O6.HCl			Topoisomerase II inhibitor, anti-neoplastic drug
Ro 31-8220 Mesylate	138489-18-6	553.65	C25H23N5O2S.CH4O3S			Pan-PKC inhibitor
Pirarubicin	72496-41-4	627.64	C32H37NO12	DNA Damage/DNA Repair	Topoisomerase	Topoisomerase II inhibitor;anthracycline antibiotic
Inauhzin	309271-94-1	469.58	C25H19N5OS2			SIRT1 inhibitor
MG 149	1243583-85-8	340.46	C22H28O3			HAT inhibitor
Nexturastat A	1403783-31-2	341.4	C19 H23 N3 O3	Chromatin/Epigenetics	HDAC	HDAC6 inhibitor,highly potent and selective
BMS-345541(free base)	445430-58-0	255.32	C14H17N5	Chromatin/Epigenetics	HDAC	IKK-1/IKK-2 inhibitor,potent and selective
CPI-169	1450655-76-1	528.66	C27H36N4O5S	Stem Cell	EZH2	EZH2 inhibitor
Santacruzamate A (CAY10683)	1477949-42-0	278.35	C15H22N2O3	DNA Damage/DNA Repair	HDAC	HDAC inhibitor, potent and selective
Procainamide HCl	614-39-1	271.79	C13H22ClN3O	Membrane Transporter/Ion Channel	Sodium Channel	sodium channel blocker
CAY10603	1045792-66-2	446.5	C22H30N4O6	DNA Damage/DNA Repair	HDAC	potent and selective inhibitor of HDAC6
PFI-2	1627676-59-8	499.52	C23H25F4N3O3S	Chromatin/Epigenetics	Histone Methyltransferase	SETD7 methyltransferase inhibitor
Remodelin	1622921-15-6	363.28	C15H15BrN4S	Chromatin/Epigenetics	Histone Acetyltransferases	NAT10 inhibitor
ML324	1222800-79-4	349.43	C21H23N3O2	Chromatin/Epigenetics	Histone Demethylases	JMJD2 demethylase inhibitor, potent and cell-permeable
SP2509	1423715-09-6	437.9	C19H20ClN3O5S	Chromatin/Epigenetics	Histone Demethylases	Demethylase 1 (LSD1) antagonist, novel Lysine-specific
OF-1	919973-83-4	440.31	C17H18BrN3O4S	Chromatin/Epigenetics	Bromodomain	BRPF1B and BRPF2 bromodomain inhibitor
GSK 5959	901245-65-6	394.47	C22H26N4O3	Chromatin/Epigenetics	Bromodomain	BRPF1 bromodomain inhibitor
A 366	1527503-11-2	329.44	C19H27N3O2	Chromatin/Epigenetics	Histone Methyltransferase	G9a/GLP histone lysine methyltransferase inhibitor
WDR5 0103	890190-22-4	383.44	C21H25N3O4	Chromatin/Epigenetics	Histone Methyltransferase	WD repeat-containing protein 5 (WDR5) antagonist
GSK-LSD1 2HCl	1431368-48-7	289.24	C14H22Cl2N2	Chromatin/Epigenetics	Histone Demethylases	irreversible, and selective LSD1 inhibitor
EPZ015666	1616391-65-1	383.44	C20H25N5O3	Chromatin/Epigenetics	Histone Methyltransferase	PRMT5 inhibitor
Mirin	1198097-97-0	220.25	C10H8N2O2S	Cell Cycle/Checkpoint	ATM/ATR	Mre11-Rad50-Nbs1 (MRN)-ATM pathway inhibitor
IOX 1	5852-78-8	189.17	C10H7NO3	Chromatin/Epigenetics	Histone Demethylases	histone demethylase JMJD inhibitor
GSK J2	1394854-52-4	389.45	C22H23N5O2	Chromatin/Epigenetics	Histone Demethylases	inactive control of GSK J1, JMJD3 (KDM6B) and UTX (KDM6A) inhibitor
G007-LK	1380672-07-0	529.96	C25H16ClN7O3S	DNA Damage/DNA Repair	tankyrase	tankyrase 1/2 inhibitor
GSK503	1346572-63-1	526.67	C31H38N6O2	Stem Cell	EZH2	EZH2 inhibitor
FG2216	223387-75-5	280.66	C12H9ClN2O4	Angiogenesis	HIF	HIF-prolyl hydroxylase inhibitor
Molidustat (BAY85-3934)	1154028-82-6	314.3	C13H14N8O2	Chromatin/Epigenetics	HIF	Novel HIF-PH inhibitor
GSK2879552	1401966-69-5	364.48	C23H28N2O2	Chromatin/Epigenetics	Histone Demethylases	Novel and irreversible LSD1 inhibitor
ORY-1001	1431326-61-2	303.27	C15H24Cl2N2	DNA Damage/DNA Repair	HDAC	Selective inhibitor of KDM1A.
BET bromodomain inhibitor	1380087-89-7	365.81	C20H16ClN3O2	Chromatin/Epigenetics	Bromodomain	Potent and selective inhibitor for BRD4
C7280948	587850-67-7	276.35	C14H16N2O2S	Chromatin/Epigenetics	Histone Methyltransferase	Novel PRMT1 inhibitor
Chidamide	743420-02-2	390.41	C22H19FN4O2	Chromatin/Epigenetics	HDAC	Novel HDAC inhibitor
Decernotinib(VX-509)	944842-54-0	392.38	C18H19F3N6O	Chromatin/Epigenetics	JAK	Selective and orally active JAK3 inhibitor
ACY-241	1316215-12-9	467.95	C24H26ClN5O3	Chromatin/Epigenetics	HDAC	Selective and orally active HDAC6 inhibitor
LFM-A13	244240-24-2	360	C11H8Br2N2O2	Angiogenesis	BTK	BTK-specific tyrosine kinase inhibitor
UF 010	537672-41-6	271.15	C11H15BrN2O	Chromatin/Epigenetics	HDAC	Novel and selective class I HDAC inhibitor
BML-210(CAY10433)	537034-17-6	339.43	C20H25N3O2	Chromatin/Epigenetics	HDAC	Novel HDAC inhibitor
2-hexyl-4-Pentynoic Acid	96017-59-3	182.26	C11H18O2	Chromatin/Epigenetics	HDAC	Potent and robust HDACs inhibitor
Triacetyl Resveratrol	42206-94-0	354.35	C20H18O6	Chromatin/Epigenetics	Sirtuin	Cell-permeable resveratrol prodrug
2,4-Pyridinedicarboxylic Acid	499-80-9	167.12	C7H5NO4	Chromatin/Epigenetics	Histone Demethylases	Histone LSD inhibitor
PFI 4	900305-37-5	380.44	C21H24N4O3	Chromatin/Epigenetics	Bromodomain	Potent and selective BRPF1 Bromodomain inhibitor
Suberohydroxamic Acid	38937-66-5	204.22	C8H16N2O4	Chromatin/Epigenetics	HDAC	HDAC inhibitor
MN 64	92831-11-3	264.32	C18H16O2	DNA Damage/DNA Repair	tankyrase	tankyrase inhibitor
GSK2801	1619994-68-1	371.45	C20H21NO4S	Chromatin/Epigenetics	Bromodomain	inhibitor of BAZ2A and BAZ2B bromodomains
PX-478 2HCl	685898-44-6	394.12	C13H20Cl4N2O3	Chromatin/Epigenetics	HIF	HIF-1α inhibitor
SirReal2	709002-46-0	420.55	C22H20N4OS2			Sirt2 inhibitor
SGC707	1687736-54-4	298.34	C16H18N4O2	Chromatin/Epigenetics	Histone Methyltransferase	PRMT3 inhibitor
AS8351	796-42-9	291.3	C17H13N3O2	Chromatin/Epigenetics	Histone Demethylases	histone demethylase inhibitor
Daprodustat(GSK1278863)	960539-70-2	393.43	C19H27N3O6	Angiogenesis	HIF	HIF-prolyl hydroxylase inhibitor
LLY507	1793053-37-8	574.76	C36H42N6O	Chromatin/Epigenetics	Histone Methyltransferase	SMYD2 inhibitor
Picolinamide	1452-77-3	122.12	C6H6N2O	DNA Damage/DNA Repair	PARP	poly (ADP-ribose) synthetase inhibitor
Benzamide	55-21-0	121.14	C7H7NO	DNA Damage/DNA Repair	PARP	poly (ADP-ribose) synthetase inhibitor
GSK591	1616391-87-7	380.48	C22H28N4O2	Chromatin/Epigenetics	Histone Methyltransferase	PRMT5 inhibitor
MS023	1831110-54-3	287.4	C17H25N3O	Chromatin/Epigenetics	Histone Methyltransferase	type I PRMTs inhibitor
BI-7273	1883429-21-7	353.41	C20H23N3O3	Chromatin/Epigenetics	Bromodomain	BRD9 bromodomain inhibitor
AGK 2	304896-28-4	434.27	C23H13Cl2N3O2	Chromatin/Epigenetics	Sirtuin	SIRT2 inhibitor, potent and selective
Tranylcypromine hydrochloride	1986-47-6	169.65	C9H11N.HCl	Chromatin/Epigenetics	Histone Demethylases	LSD1/BHC110 and monoamine oxidase (MAO) inhibitor
(R)-(+)-Etomoxir sodium salt	828934-41-4	320.74	C15H18ClNaO4	Chromatin/Epigenetics	Histone Acetyltransferases	carnitine palmitoyltransferase I (CPT1) inhibitor
PFI 3	1819363-80-8	321.37	C19H19N3O2	Chromatin/Epigenetics	Bromodomain	inhibitor of polybromo 1 and SMARCA4
UNC 0642	1481677-78-4	546.7	C29H44F2N6O2	Chromatin/Epigenetics	Histone Methyltransferase	G9a and GLP histone lysine methyltransferase inhibitor
CPI-637	1884712-47-3	386.45	C22H22N6O	Chromatin/Epigenetics	Bromodomain	CBP/EP300 bromodomain inhibitor
ITSA-1 (ITSA1)	200626-61-5	292.12	C13H7Cl2N3O	Chromatin/Epigenetics	HDAC	HDAC activator via Trichostatin A (TSA) suppression
Thiomyristoyl	1429749-41-6	581.37	C34H51N3O3S			
Cerdulatinib (PRT062070)	1198300-79-6	445.54	C20H27N7O3S	Chromatin/Epigenetics	JAK	Syk/JAK inhibitor
PTP Inhibitor II	2632-13-5	229.1	C9H9BrO2	Chromatin/Epigenetics	Protein Ser/Thr Phosphatases	protein tyrosine phosphatase (PTP) inhibitor
PTP Inhibitor I	2491-38-5	215	C8H7BrO2	Chromatin/Epigenetics	Protein Ser/Thr Phosphatases	tyrosine phosphatase (PTP) inhibitor
4-iodo-SAHA	1219807-87-0	390.2	C14H19IN2O3	Chromatin/Epigenetics	HDAC	class I and class II histone deacetylase (HDAC) inhibitor
β-Glycerophosphate (sodium salt hydrate)	13408-09-8	306.1	C3H7O6P ? 2Na [5H2O]	Chromatin/Epigenetics	Protein Ser/Thr Phosphatases	protein phosphatase inhibitor
Parthenolide	20554-84-1	248.32	C15H20O3	Immunology/Inflammation	NF-κB	
6-gingerol	23513-14-6	294.39	C17H26O4	Nature Products		
Nicotinamide	98-92-0	122.13	C6H6N2O	Nature Products		PARP-1 inhibitor
Coumarin	91-64-5	146.14	C9H6O2	Nature Products		Precursor in chemical reaction
Diosgenin	512-04-9	414.61	C27H42O3	Nature Products		

Curcumol	4871-97-0	236.35	$C_{15}H_{24}O_2$	Nature Products		
Quercetin	117-39-5	302.24	$C_{15}H_{10}O_7$	PI3K/Akt/mTOR Signaling	PI3K	Antitumor agent
Ginkgolide C	15291-76-6	440.4	$C_{20}H_{24}O_{11}$	Nature Products		
Fisetin	528-48-3	286.05	$C_{15}H_{10}O_6$	Nature Products		
